# Deep mining reveals the diversity of endogenous viral elements in vertebrate genomes

**DOI:** 10.1038/s41564-024-01825-4

**Published:** 2024-10-22

**Authors:** Jose Gabriel Nino Barreat, Aris Katzourakis

**Affiliations:** Department of Biology, University of Oxford, Oxford, UK

**Keywords:** Evolution, Ecology

## Abstract

Integration of viruses into host genomes can give rise to endogenous viral elements (EVEs), which provide insights into viral diversity, host range and evolution. A systematic search for EVEs is becoming computationally challenging given the available genomic data. We used a cloud-computing approach to perform a comprehensive search for EVEs in the kingdoms *Shotokuvirae* and *Orthornavirae* across vertebrates. We identified 2,040 EVEs in 295 vertebrate genomes and provide evidence for EVEs belonging to the families *Chuviridae*, *Paramyxoviridae*, *Nairoviridae* and *Benyviridae*. We also find an EVE from the *Hepacivirus* genus of flaviviruses with orthology across murine rodents. In addition, our analyses revealed that reptarenaviruses and filoviruses probably acquired their glycoprotein ectodomains three times independently from retroviral elements. Taken together, these findings encourage the addition of 4 virus families and the *Hepacivirus* genus to the growing virus fossil record of vertebrates, providing key insights into their natural history and evolution.

## Abstract (web summary)

Computational cloud-based screen of vertebrate genomes identifies endogenous viral elements of members of the kingdoms *Shotokuvirae* and *Orthornavirae* and informs about evolutionary history of non-retroviral elements.

## Main

Viruses of all genome types can potentially integrate into host genomes and give rise to endogenous viral elements (EVEs)^1^. An EVE forms when viral genetic information enters the host germline and is transmitted vertically to offspring. A novel EVE exists initially as an insertion polymorphism but can eventually reach fixation subject to the forces of natural selection and genetic drift^1^. These fixed EVEs have the highest chance of surviving long periods of time in host genomes and therefore provide valuable information on virus–host associations over geological timescales. In particular, discovery of endogenous viruses can expand both taxonomic and biogeographical host range, as well as establish direct timelines of association between virus and host^2,3^. Therefore, EVEs constitute a genomic fossil record that preserves information on ancient viruses and their interactions.

Although endogenous retroviruses are the most abundant type of EVE found in vertebrate genomes, multiple EVEs of non-retroviral origin have been described. Currently, these can be assigned to 5 viral kingdoms: *Pararnavirae* (*Hepadnaviridae*)^4^, *Heunggongvirae* (*Herpesviridae* and *Teratorns*)^5,6^, *Bamfordvirae* (*Mavericks*/*Polintons*)^7^, *Shotokuvirae* (*Parvoviridae* and *Circoviridae*)^8,9^ and *Orthornavirae* (*Bornaviridae*, *Filoviridae* and *Flaviviridae*)^10–12^. EVEs from the kingdoms *Shotokuvirae* and *Orthornavirae* are among the most abundant and diverse non-retroviral EVEs. The kingdom *Shotokuvirae* comprises 16 families of single-stranded DNA (ssDNA) and double-stranded DNA (dsDNA) viruses that descended from an ancestral HUH (histidine-hydrophobic-histidine endonuclease)-encoding virus^13,14^. The kingdom *Orthornavirae* comprises 112 families of RNA viruses that encode the RNA-dependent RNA polymerase (RdRp)^14^. Both shotokuviruses and orthornaviruses include members that are pathogenic to vertebrates. For example, in parrots (Psittacidae), the circovirus beak and feather disease virus can cause immunosuppression and loss of feathers, with potentially fatal outcomes^15^. Canine parvovirus is highly contagious and can cause serious illness in domestic and wild canids^16^. In the kingdom *Orthornavirae*, viruses from the families *Filoviridae*, *Arenaviridae* and *Nairoviridae* can cause haemorrhagic fevers with high case fatality rates (up to 30–90%) in humans^17–19^. Additional orthornaviruses in the families *Paramyxoviridae* (mumps, measles and parainfluenza viruses)^20–22^ and *Flaviviridae* (yellow fever, dengue and Zika viruses)^23^ are also major contributors to human disease.

We took advantage of the larger sequence data sets available today together with a cloud-computing approach to carry out a comprehensive search for EVEs from the kingdoms *Shotokuvirae* and *Orthornavirae* in vertebrate genomes. We chose cloud-computing over other methods since it enables large-scale searches of public NCBI databases, with a minimal requirement for computational resources from the user. They can also take advantage of highly scalable containerized runtime environments (for example, kubernetes), and storage needs can also be outsourced to the cloud. Using this strategy, we reveal thousands of EVEs, many belonging to viral families that had not been found previously. In addition, our results shed light on the evolutionary history and ecology of multiple viral lineages, and highlight the value of cloud computing for characterizing the diversity of EVEs in vertebrate genomes.

## Results

We conducted a comprehensive search for EVEs in the kingdoms *Shotokuvirae* and *Orthornavirae* using a recently developed cloud-based tool (ElasticBLAST) on the Google Cloud Platform (https://cloud.google.com). This allowed us to efficiently query the representative vertebrate genomes database (ref_euk_rep_genomes, taxid: ‘7742’) with 24,478 viral protein queries. We identified a total of 2,040 EVEs in the genome assemblies of 295 vertebrates, in addition to 17 exogenous virus sequences (Supplementary Figs. 1 and 2 and Data). These include EVEs belonging to the families *Chuviridae* (121 EVEs), *Paramyxoviridae* (19 EVEs), *Benyviridae* (22 EVEs), *Nairoviridae* (1 EVE) and an EVE from the *Hepacivirus* genus of flaviviruses. We also identified close hits to the ectodomains of reptarenaviruses in tarsier genomes and to the ectodomains of filoviruses in the genomes of cartilaginous fish and the Komodo dragon contained within retrovirus-like elements, which suggest a macroevolutionary scenario for the origin of glycoprotein ectodomains.

Bornavirus, parvovirus and circovirus EVEs were found in hosts that expand the range of these families to amphibians, lungfish, coelacanths and egg-laying mammals. We found nucleoprotein and glycoprotein-like EVEs from bornaviruses in the common toad (*Bufo bufo*) and Chusan Island toad (*Bufo gargarizans*), and bornavirus L polymerase-like EVEs in the West African lungfish (*Protopterus annectens*). A VP1-like parvovirus EVE was found in the west Indian Ocean coelacanth (*Latimeria chalumnae*) and an NS1-like parvovirus EVE in the Gaboon caecilian (*Geotrypetes seraphini*). Rep-like EVEs from circoviruses were detected in the genome of the Gaboon caecilian (*Geotrypetes seraphini*), and capsid- and Rep-like circovirus EVEs in the short-beaked echidna (*Tachyglossus aculeatus*).

### Chuvirus EVEs in fish, mammals and non-avian reptiles

**Fig. 1:**
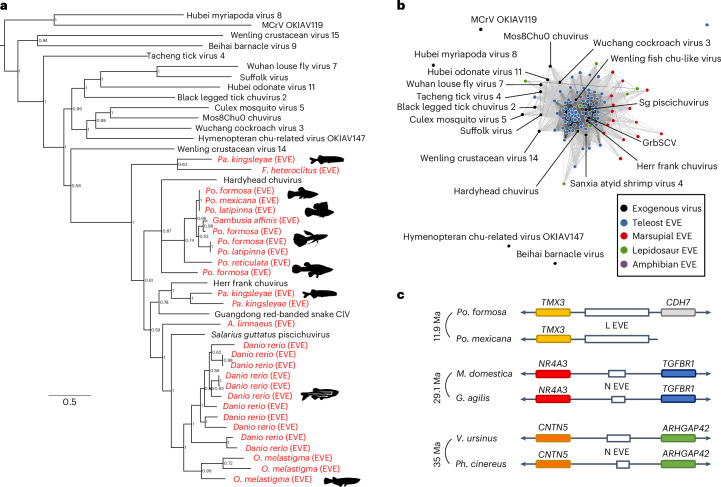
Chuvirus EVEs in vertebrate genomes. **a**, Bayesian phylogenetic tree of the RdRps of exogenous chuviruses and the EVEs found in teleost fish (in red). Some species have multiple integrations suggesting a close interaction with these viruses and recurrent integrations, or possibly a single integration event followed by intragenomic amplification. Note how the vertebrate-associated viruses form a monophyletic group, while the arthropod-associated ones are paraphyletic. The tree was rooted with Hubei myriapoda virus 8 (*Myriaviridae*) and Megalopteran chu-related virus 119 (MCrV, *Crepuscoviridae*) as outgroups. Tree inferred in MrBayes3 using the LG + F + I + G4 model and 4.74 M generations (relative burn-in = 25%). EVEs are shown in red. *Pa*., *Paramormyrops*; *F*., *Fundulus*; *Po*., *Poecilia*; *A*., *Austrofundulus*; *O*., *Oryzias*; ClV, chuvirus-like virus. **b**, CLANS network of the nucleoprotein of exogenous chuviruses, vertebrate chuvirus EVEs and the two outgroups mentioned above. Edges are drawn between nodes with a significance of *P* < 1 × 10^−15^. The vertebrate EVEs are well connected to the central network that includes vertebrate-associated chuviruses and a number of chuviruses from arthropods. *Sg*, *Salarius guttatus*; GrbSCV, Guangdong red-banded snake chuvirus-like virus. **c**, Syntenic arrangement of the most-proximal genes was used to establish orthology of three integrations. The minimum date of integrations in each species pair is based on the divergence of the host species in TimeTree. *M*., *Monodelphis*; *G*., *Gracilinanus*; *V*., *Vombatus*; *Ph*., *Phascolarctos*. Source data

Chuviruses are negative-sense RNA viruses (Order *Jingchuvirales*) described mainly from metagenomic samples^24^. They have been found in arthropods and associated with a number of vertebrates^24^. Chuvirus-like EVEs have been described in a number of arthropod genomes^25,26^. We found 28 EVEs similar to the RNA-dependent RNA polymerase in teleost fish and 92 EVEs similar to the nucleoprotein in teleosts, amphibians, snakes and lizards, and marsupials (Fig. 1). The vertebrate-associated chuviruses form a well-supported clade with the chuvirus EVEs (posterior probability = 1) in the RdRp phylogeny (Fig. 1a) and are well connected to chuvirus EVEs found in vertebrates in the nucleoprotein network (Fig. 1b). Examination of EVE loci from teleosts and marsupials revealed that some of these integrations are orthologous and date back to 11.9–35 million years ago (Ma).

### Paramyxovirus EVEs in the genomes of teleost fish

**Fig. 2:**
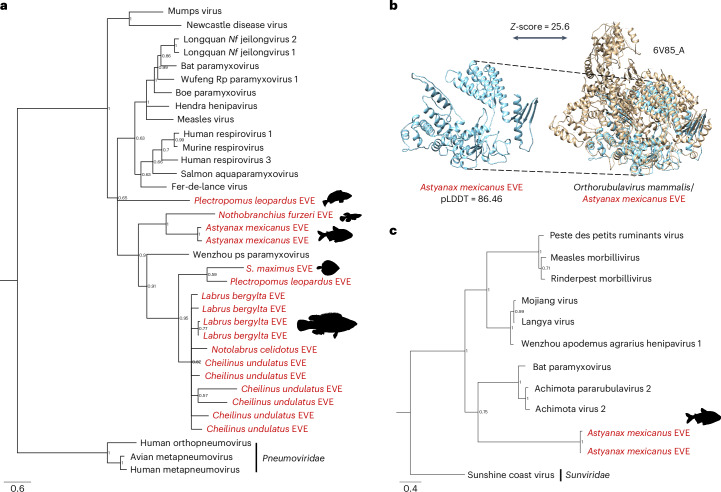
Paramyxovirus EVEs in the genomes of teleost fish. **a**, Bayesian tree of the RdRp of exogenous paramyxoviruses and the EVEs found in teleost fish (in red). Most EVEs form a clade together with Wenzhou Pacific spadenose shark paramyxovirus. The tree was outgroup rooted with RdRp sequences from pneumoviruses. Tree inferred in MrBayes3 using the LG + F + I + G4 model and 9.06 M generations (relative burn-in = 25%). EVEs are shown in red. **b**, Predicted structure of an RdRp fragment present in the genome of the Mexican tetra and comparison to the RdRp structure of parainfluenza virus 5 (*Orthorubulavirus mammalis*). pLDDT, predicted local distance difference test metric. **c**, Bayesian tree of the nucleoprotein of paramyxoviruses and the EVEs found in the Mexican tetra. The EVEs are nested within the *Paramyxoviridae* with high support (posterior probability = 1) and are closest to a group of bat paramyxoviruses with a posterior probability = 0.75. Tree inferred in MrBayes3 using the LG + I + G4 model and 1 M generations (relative burn-in = 25%). EVEs are shown in red. *Nf*, *Niviventer fulvescens*; ps, Pacific shark; *S*., *Scophthalmus*. Source data

Paramyxoviruses are non-segmented, negative-sense RNA viruses classified in the Order *Mononegavirales*^27^. Although paramyxoviruses infect a wide variety of vertebrates^27^, EVEs from paramyxoviruses had not been described. We found 17 EVEs similar to the RdRp of paramyxoviruses and 2 EVEs similar to the nucleoprotein in the genomes of teleost fish. Multiple integrations were found in species of fish from the family Labridae (*Labrus*, *Notolabrus*, *Cheilinus*), in the leopard coral grouper *Plectropomus leopardus* (Serranidae) and in the Mexican tetra *Astyanax mexicanus* (Characidae). Phylogenetic analysis placed most of the RdRp EVEs in a clade with Wenzhou Pacific spadenose shark paramyxovirus (posterior probability = 0.9), while a single EVE from the coral grouper was placed between this clade and a clade composed of paramyxoviruses such as measles virus, Hendra virus or human respiroviruses (Fig. 2a). Structural comparison of an open reading frame fragment found in the genome of the Mexican tetra to *Orthorubulavirus mammalis* revealed a conserved structure of the RdRp (Fig. 2b).

### Benyvirus-like EVEs in vertebrate genomes

**Fig. 3:**
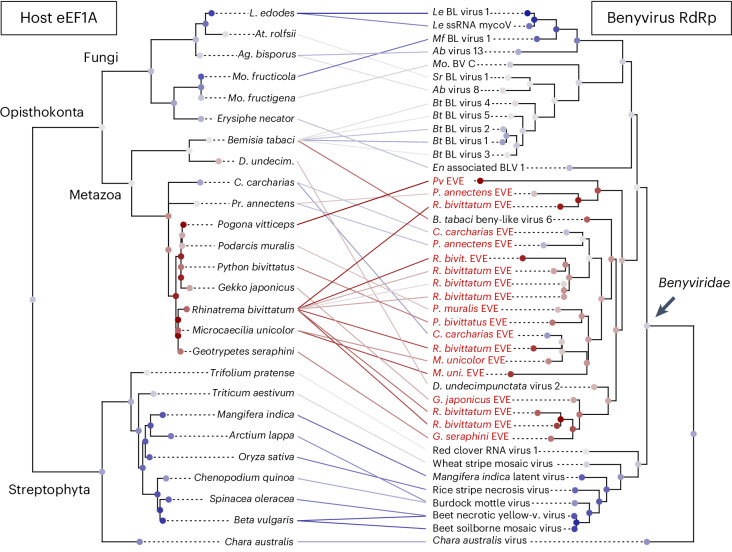
Tanglegram of the phylogenies of benyviruses (including vertebrate EVEs) and their eukaryotic hosts. The benyvirus RdRp and host eEF1A (eukaryotic translation elongation factor 1-alpha) phylogenies point at deep codivergences and more recent cross-species transmissions in the three main groups (plant, fungi, animal benyviruses). The position of *Chara australis* virus in the RdRp phylogeny (see Supplementary Fig. 3) could be interpreted as an ancient virus jump between photosynthetic organisms and the ancestors of animals and fungi (Opisthokonta). The maximum-likelihood trees were inferred in RAxML-NG (eEF1A: LG + I + G4, RdRp: LG + F + I + G4) and the tanglegram inferred using the maximum incongruence algorithm (MIC) in Rtapas. EVEs are shown in red. *L*., *Lentinula*; *At*., *Athelia*; *Ag*., *Agaricus*; *Mo*., *Monilinia*; *D*., *Diabrotica*; *C*., *Carcharodon*; *P./Pr*., *Protopterus*; *M*., *Microcaecilia*; *Le*, *Lentinula edodes*; *Mf*, *Monilinia fructicola*; *Ab*, *Agaricus bisporus*; *Sr*, *S. rolfsii*; *B.*, *Bemisia*; *Bt*, *Bemisia tabaci*; *En*, *Erysiphe necator*; BL, beny-like. Source data

Benyviruses are multipartite, positive-sense RNA viruses known to infect plants^28^, but they have also been isolated from fungi and some insects^29^. We found 19 EVEs with similarity to the RdRp of benyviruses in the genomes of caecilians (*Rhinatrema*, *Microcaecilia*), lizards (*Podarcis*, *Gekko*), snakes (*Python*), the West African lungfish (*Protopterus annectens*) and the great white shark (*Carcharodon carcharias*). In the phylogeny of benyvirus RdRps (Fig. 3), the EVEs of vertebrates were placed in a clade with two benyviruses isolated from insects (Diabrotica undecimpunctata virus 2 and Bemisia tabaci beny-like virus 6), forming a clade of animal viruses. The phylogeny also recovered a clade of benyviruses that infect land plants and another that infects mostly fungi (except for some viruses isolated from the silverleaf whitefly, *Bemisia tabaci*). A tanglegram of the benyvirus RdRps and the host phylogeny was able to recover the split between land plants and fungi + animals (Opisthokonta). In the animal-infecting group, the inconsistency of both phylogenies suggests a history of cross-species transmissions (Fig. 3). We also found 6 EVEs with similarity to the coat protein of benyviruses in lizards (*Podarcis*, *Lacerta*, *Zootoca*) and the small-spotted catshark (*Scyliorhinus canicula*).

### Nairovirus EVE in the genome of the Etruscan shrew

**Fig. 4:**
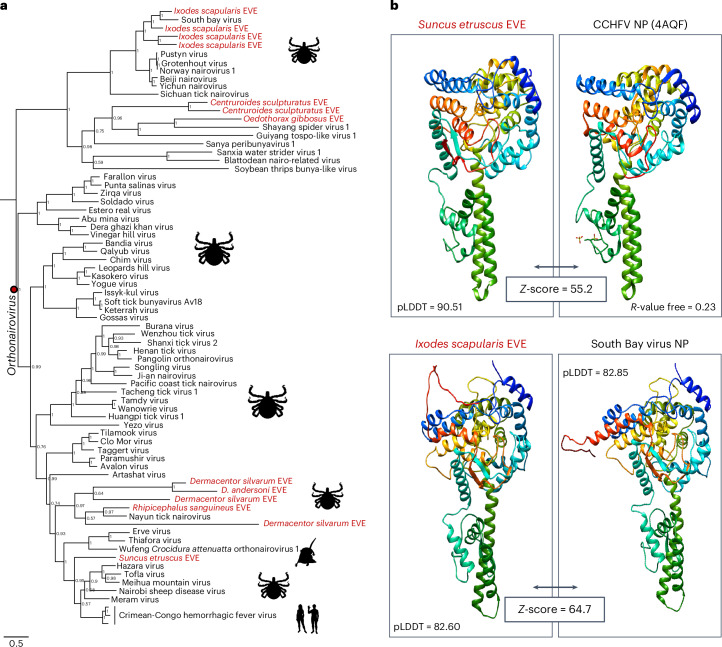
Nairovirus EVEs in the genome of the Etruscan shrew and ticks. **a**, Bayesian phylogeny of the nairovirus nucleoprotein gene including EVEs from the Etruscan shrew, ticks and other chelicerates, together with exogenous nairoviruses. The element found in the Etruscan shrew genome forms a clade with the Crimean–Congo hemorrhagic fever viruses/Hazara virus, sister to the Erve/Thiafora and Wufeng Crocidura attenuatta orthonairovirus 1 clade, known to infect soricid shrews of the subfamily Crocidurinae. The node in red shows the clade of viruses that contains recognized members of the *Orthonairovirus* genus. Tree inferred in MrBayes3 with a codon-partitioned model (1st and 3rd positions: GTR + G4, 2nd position: GTR + I + G4) and 5 M generations (relative burn-in = 25%). EVEs are shown in red. **b**, Structural comparison of nucleoproteins from EVEs in the Etruscan shrew and black-legged tick genomes with exogenous nairoviruses. Structures were modelled in Alphafold2 to a good backbone accuracy (pLDDT > 80) or downloaded from the Protein Data Bank. The Etruscan shrew element adopts a structure highly similar to the structure of Crimean–Congo hemorrhagic fever virus determined by X-ray crystallography. The black-legged tick predicted structure is more similar to the South Bay virus structure as predicted from phylogenetic analysis. Source data

Nairoviruses are negative-sense RNA viruses with 3 genomic segments S, M and L. The S segment carries the gene that encodes the nucleoprotein^30^. Nairoviruses infect arthropods and can be transmitted to humans via tick bites^30^. Some nairoviruses can cause disease in humans, but the Crimean–Congo haemorrhagic fever (CCHF) viruses are highly pathogenic^31^. Previously, EVEs similar to the nucleoprotein of nairoviruses were found in the genome of the black-legged tick *Ixodes scapularis*^1^. However, they were distantly related to the nucleoproteins of CCHF viruses. We found an EVE in the genome of the Etruscan shrew (*Suncus etruscus*), which can be placed in the same genus as CCHFV, *Orthonairovirus* (Fig. 4a). Using this sequence to query the non-redundant (nr) protein database (NCBI), we were able to identify additional orthonairovirus EVEs in the genomes of ticks (*Rhipicephalus sanguineus*, *Dermacentor silvarum*, *D. andersoni*). Comparison of the predicted EVE protein structures shows the high similarity between the nucleoproteins from the Etruscan shrew EVE and CCHFV, and between the black-legged tick and South Bay virus Ns (Fig. 4b).

### Hepacivirus EVE in the genomes of murine rodents

**Fig. 5:**
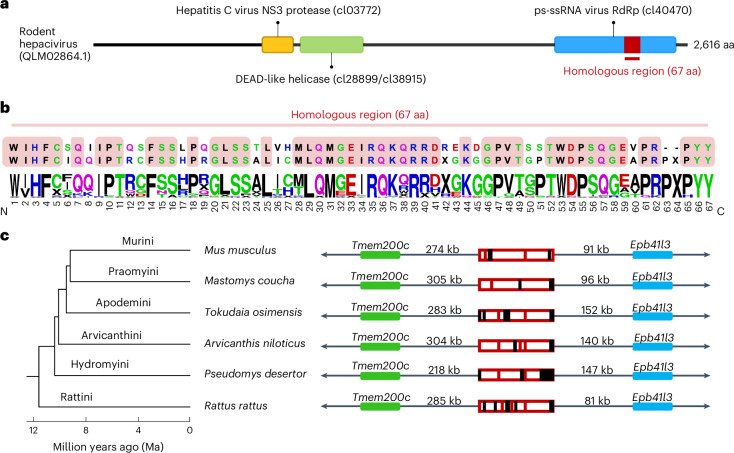
Hepacivirus EVE in the genomes of rodents from the subfamily Murinae. **a**, Conserved domain annotation of the rodent hepacivirus ETH674/ETH/2012 (QLM02864.1) polyprotein. The region of homology to the EVEs is embedded within the ps-ssRNA domain. **b**, Comparison of the region of homology between rodent hepacivirus ETH674/ETH/2012 (top sequence) and the consensus sequence obtained from 21 murine genomes (middle sequence). Identical amino acids at a given position are highlighted in a red box (the two sequences are 75% pairwise identical at the amino acid level). The sequence logo at the bottom shows variation at the given position proportional to frequency (0–100%). **c**, Orthology across 6 representative species in 6 tribes (Murini, Praomyini, Apodemini, Arvicanthini, Hydromyini, Rattini) of the subfamily Murinae, together with a phylogeny of the group. Flanking genes were identified in the mouse (*Mus musculus*) assembly and used to annotate the region in the other assemblies. Red bars, internal stop codons; black rectangles, indel mutations. Source data

Hepaciviruses are positive-sense RNA viruses in the family *Flaviviridae*, which are classified in the genus *Hepacivirus*^32^. People chronically infected with hepatitis C virus (HCV) are at a substantial risk of liver disease including fibrosis, cirrhosis and hepatocellular carcinoma^33^. We found hits homologous to a ~67-amino acid (aa) fragment of the positive-sense single-stranded RNA polymerase domain (Superfamily cl40470) of rodent hepacivirus ETH674/ETH/2012 in the genomes of rodents in the subfamily Murinae (Fig. 5a,b). Examination of the genomic context across 21 species showed that the integration was orthologous but degraded in murine genomes (Fig. 5c). Given that the hepacivirus EVE is shared between mice (*Mus* spp.) and rats (*Rattus* spp.), this suggests a minimum age of 11.7–14.2 Ma^34^. So far, we have been able to identify this insertion only in the polymerase domain of rodent hepacivirus ETH674/ETH/2012 isolated from the Ethiopian white-footed mouse (*Stenocephalemys albipes*). Nonetheless, the C-terminal end of the EVE consensus sequence [**QGE**A**PR**(PX)**PYY**] shares homology with a conserved sequence found in the polymerases of many other exogenous hepaciviruses [**QGE**V**PRPYY**], further suggesting this is in fact an EVE of hepaciviral origin.

### Origin of the ectodomain in filoviruses and reptarenaviruses

**Fig. 6:**
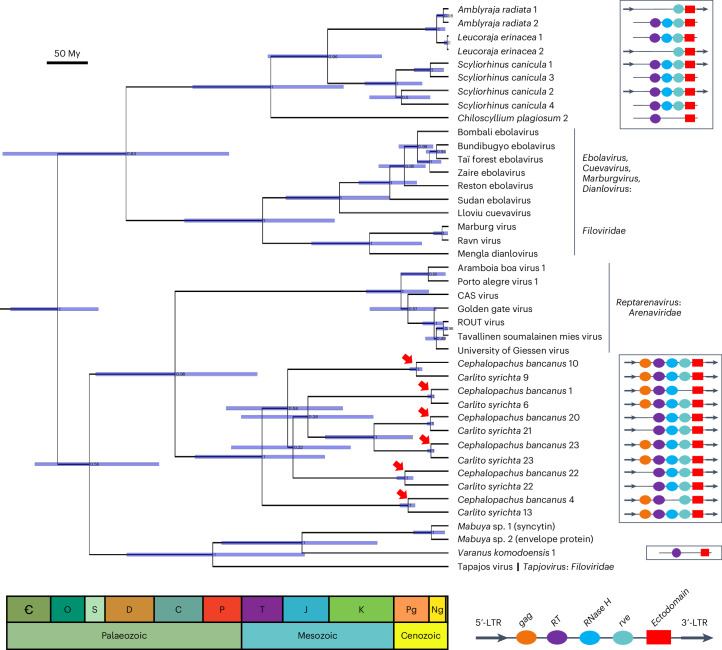
Bayesian timetree of the ectodomain homologues found in retroviruses, filoviruses and reptarenaviruses. The ectodomains of reptarenaviruses form a highly supported clade (posterior probability = 0.98) with the endogenous ectodomains found in tarsiers (*Carlito syrichta*, *Cephalopachus bancanus*). The ectodomains of ebolaviruses, cuevaviruses, marburgviruses and dianloviruses form a clade which is the sister group to the endogenous ectodomains found in cartilaginous fish. However, the ectodomain of Tapajos virus forms a distinct clade (posterior probability = 1) with endogenous ectodomains found in lizards (*Mabuya*, *Varanus*), suggesting that the Tapajos virus ectodomain was captured independently from the ectodomains of other filoviruses. The tree was inferred in BEAST2 with the JTT + G4 site model using the optimized relaxed clock (ORC) and 20 M generations (relative burn-in = 25%). The red arrows indicate pairs of tarsier orthologues. A diagram with the genomic context of the endogenous ectodomains is shown to the right and suggests that the endogenous ectodomains form part of endogenous retroviral elements. C, Cambrian; O, Ordovician; S, Silurian; D, Devonian; C, Carboniferous; P, Permian; T, Triassic; J, Jurassic; K, Cretaceous; Pg, Paleogene; Ng, Neogene. Source data

The envelope proteins of retroviruses and the glycoproteins of some filoviruses (*Ebolavirus*, *Marburgvirus*, *Cuevavirus*, *Dianlovirus* and *Tapjovirus*) contain an ectodomain with heptad-repeat sequences and an immunosuppressive domain (ISD) region^35^. The glycoproteins of arenaviruses in the genus *Reptarenavirus* also contain a similar ectodomain^36^. We found hits closely related to the ectodomain of reptarenaviruses in the genomes of the Philippine and the western tarsier (*Carlito syrichta* and *Cephalopachus bancanus*, respectively) (Supplementary Data). These hits were in close proximity to other retroviral domains (gag, RT, RNaseH, rve), were flanked by direct repeats and occurred at the expected relative position of the *env* gene, establishing that they were in retroviral elements. By searching for other sequences related to filovirus and reptarenavirus ectodomains, we found additional hits surrounded by retroviral features (or annotated as such) in the genomes of lizards (*Mabuya*, *Varanus*) and cartilaginous fish (*Chiloscyllium*, *Scyliorhinus*, *Amblyraja*, *Leucoraja*). After confirming that additional retrovirus ectodomains fell outside this clade (Supplementary Fig. 4), we focused on the ingroup to construct a time-calibrated tree (Fig. 6).

In the Bayesian phylogeny (Fig. 6), the ectodomains of reptarenaviruses were placed with high confidence (posterior probability = 0.98) as the sister group to the ectodomains in tarsiers. The ectodomains from ebola-, cueva-, marburg- and dianloviruses were placed as the sister clade to the ectodomains of retroelements found in cartilaginous fish (posterior probability = 0.83). On the other hand, the ectodomain from the filovirus Tapajos virus (*Tapjovirus*), which was found in the venom gland of the common lancehead viper (*Bothrops atrox*)^37^, was placed forming a strongly supported clade with ectodomains found in lizard retroelements (posterior probability = 1). These findings suggest that ectodomains have been captured from retroviral elements 3 times independently, twice by filoviruses and once by reptarenaviruses, over a timescale of hundreds of millions of years. We believe that the alternative scenario of a single ectodomain gain in filoviruses followed by two losses in the fish filovirus genera (*Striavirus* and *Thamnovirus*/*Oblavirus*) is inconsistent with the position of the Tapajos virus ectodomain outside the clade formed by other filoviruses.

### Evidence of purifying selection acting on 33 orthogroups

We detected 145 orthogroups in our systematic search for orthology, comprising a total of 1,114 EVEs. We found 48 orthogroups from EVEs in the family *Bornaviridae* (birds, mammals), 6 in the family *Chuviridae* (fish, snakes), 26 in the family *Filoviridae* (mammals), 50 in the family *Parvoviridae* (birds, snakes and mammals), 1 orthogroup in the genus *Hepacivirus* (rodents) and 14 orthogroups in the family *Circoviridae* (birds, fish and mammals). Most estimated ages of orthogroups ranged from 6.4 (5.9–7.6, adjusted time: 7.2) million years (Myr) for a parvovirus VP orthologue found in the house mouse (*Mus musculus*) and Gairdner’s shrewmouse (*Mus pahari*), to 81 (68.5–82.6, adjusted time: 81) Myr for 2 parvovirus Rep elements, a parvovirus VP and a bornavirus N found in the clade Scrotifera (Mammalia: Laurasiatheria)^34^. We found another ancient element, a circovirus Rep orthologue from cyprinid fish (*Sinocyclocheilus grahami* and *Megalobrama amblycephala*) with an estimated age of 106 Myr; however, the adjusted time estimate for this divergence is 52 Myr^34^. Overall, the ages of the oldest orthogroups we found for the families *Bornaviridae* (~94 Myr), *Parvoviridae* (~81 Myr), *Circoviridae* (~62 Myr) and *Filoviridae* (~53 Myr) are consistent with previous findings^9,10,38,39^.

We found evidence of significant purifying selection (d*N*/d*S* < 1, *P* < 0.05; acting on open reading frames (ORFs) ≥100 amino acids) in 33 of the 145 orthogroups. Purifying selection was detected in: 3 bornavirus N orthogroups (Murinae, *Myotis*, Simiiformes), 1 bornavirus G orthogroup (Fereuungulata), 9 bornavirus L orthogroups (*Myotis* × *5*, Yangochiroptera, Vespertilionidae, Murinae, Australidelphia), 3 filovirus N orthogroups (Vespertilionidae, Diprotodontia, Arvicolinae), 1 chuvirus L orthogroup (*Poecilia*), 5 circovirus Rep orthogroups (Carnivora, Caniformia, Cyprinidae, Polypteridae, Salmoninae), 4 parvovirus Rep orthogroups (Euaustralidelphia, Scrotifera, Vespertilionidae, *Thamnophis*) and 7 parvovirus VP orthogroups (Euungulata, Australidelphia, Diprotodontia × 2, Phyllostomidae × 2, Passeri) (Supplementary Data). In some of these groups, purifying selection was detected in the ORFs of some but not all species (free ratio model favoured). We also noticed 17 large ORFs (>400 amino acids) among the set of orthogroups (Supplementary Data).

## Discussion

We discovered EVEs in vertebrate genomes belonging to the families *Chuviridae*, *Paramyxoviridae*, *Benyviridae* and *Nairoviridae*. This represents the addition of 4 non-retroviral families to the 9 previously found in the genomic fossil record of vertebrates^1,5,7,12^. We also identified a *Hepacivirus* EVE in the genomes of murine rodents and found retroviral elements with ectodomains related to those of reptarenaviruses and filoviruses. Endogenous viral elements in the families *Circoviridae*, *Parvoviridae*, *Bornaviridae, Filoviridae* and *Flaviviridae* accounted for 91% of the EVEs (1,858/2,040) found during our search. Therefore, in a single systematic search, our strategy allowed for both increased sensitivity as well as reproduction of previous findings in the field.

Chuviruses are a family of RNA viruses found in arthropod-associated metagenomes, as well as EVEs in the genomes of arthropods^24,26^. A number of chuviruses have also been found associated with metagenomes from vertebrates^40^. We show evidence that chuviruses actively infect vertebrates by the discovery of 121 EVEs in teleost fish, lepidosaurs, amphibians and marsupials. The vertebrate-associated chuviruses formed a clade with the chuvirus EVEs in vertebrates (posterior probability = 1), strongly supporting that there is a vertebrate-specific clade of chuviruses. The detection of orthology of several chuvirus EVEs on the order of 11–35 Ma indicate that chuviruses have infected vertebrates from at least the Eocene epoch. These results are in line with recent evidence that chuviruses can infect and cause lymphocytic meningoencephalomyelitis in turtles^40^.

We found 22 vertebrate EVEs that could be assigned to the family *Benyviridae*. Benyviruses are plant pathogens, but a few viruses have been identified from insect metagenomes^41,42^. Our study uncovered endogenous benyviruses in vertebrate genomes, which form an animal-specific clade with four benyviruses isolated from insects (*Diabrotica undecimpunctata*, *Sesamia inferens* and *Harmonia axyridis*, Supplementary Fig. 3). This implies that a clade of benyviruses exhibits tropism for hosts in the kingdom Animalia. As shown in Fig. 3, the benyviruses of animals seem to undergo frequent cross-species transmissions. In addition, we uncovered 19 EVEs from paramyxoviruses in both freshwater and marine teleost fish. Paramyxoviruses are known to infect fish^43^, and some have been associated with disease including epidermal/gill necrosis, gill inflammation and buccal/opercular haemorrhage^44^. Our results highlight the need to better characterize the diversity of paramyxoviruses in fish hosts.

We provide evidence for an EVE from the genus *Hepacivirus* in murine rodents. This EVE shares high homology (75% amino acid identity) across a segment of the polymerase domain with rodent hepacivirus ETH674/ETH/2012. Further confirmation of orthology across rodents of the Murinae subfamily constitute direct evidence that hepaciviruses have infected murine rodents for at least 11.7–14.2 Myr. Rodents in the subfamily Murinae are inferred to have shared a most recent common ancestor in Southeast Asia 15.9 (14.1–18.2) Ma^45^, while the sequence of rodent hepacivirus ETH674/ETH/2012 was isolated from an Ethiopian white-footed mouse (*Stenocephalemys albipes*)^46^, suggesting a close coevolutionary history with murine rodents. These observations agree with recent findings that highlight murid rodents as important hepacivirus hosts^46,47^. They also agree with molecular estimates based on present-day sequences that have taken into account the time-dependent rate phenomenon (TDRP)^47^ and which suggests an origin of the *Hepacivirus* genus at least ~22 Ma^47^. Given that the homologous sequence found in rodent hepacivirus ETH674/ETH/2012 and the murine rodent EVE seems to be a unique derived feature (synapomorphy), it appears likely that hepaciviruses as a whole are older than 22 Myr.

Although nairovirus-like EVEs had been described in black-legged ticks (*Ixodes scapularis*)^1^, we identified a vertebrate orthonairovirus EVE in the genome of the Etruscan shrew (*Suncus etruscus*). This EVE is the closest to the clade which includes the Crimean–Congo hemorrhagic fever viruses. Discovery of this element points to the importance of shrews as reservoirs of potentially pathogenic orthonairoviruses. The related Erve and Thiafora viruses found in France and Senegal, were initially isolated from shrews (*Crocidura russula*, *Crocidura* sp.)^48,49^. A number of recently discovered orthonairoviruses have also been isolated from shrews including: Wufeng orthonairovirus 1 from *Crocidura attenuata* in China, Lamusara and Lamgora viruses from *Crocidura goliath* in Gabon^50^, and Cencurut virus from *Suncus murinus* in Singapore^51^. These data indicate that shrews in the subfamily Crocidurinae are important natural reservoirs of orthonairoviruses in Europe, Africa and Asia. Our discovery of EVEs related to Nayun tick nairovirus in *Rhipicephalus sanguineus*, *Dermacentor andersoni* and *D. silvarum* implicate these tick species as additional vectors of orthonairoviruses. This agrees with the isolation of Nayun tick nairovirus from a *Rhipicephalus* tick^52^. These observations suggest a close interaction between multiple tick species with nairoviruses and support the role of crocidurine shrews as important reservoirs of orthonairoviruses.

There is potential for non-retroviral EVEs to function in EVE-derived immunity. In the thirteen-lined squirrel (*Ictidomys tridecemlineatus*), an endogenous bornavirus-like N gene (416-aa long) can inhibit Borna disease virus (BDV) replication and block de novo infection by BDV^53^. Recently, a parvoviral-like Rep gene in the genome of degus (*Octodon degus*), encoding a 508-aa product, was shown to inhibit replication of the model parvovirus minute virus of mice (MVM)^54^. We found 17 large open reading frames (>400 amino acids) in our orthogroups, which show similarity to parvovirus Rep/VP, chuvirus N/L and bornavirus L protein. Although not found in orthogroups, we also noticed large open reading frames in paramyxovirus EVEs (N/L-like, 3 EVEs) and the orthonairovirus N EVE found in the Etruscan shrew (484 amino acids). This possibility is supported by the evidence of significant purifying selection that we found in 33 orthogroups in 20 host clades. These comprised EVEs similar to bornavirus N/G/L, filovirus N, chuvirus L, circovirus Rep and parvovirus Rep/VP. However, it is possible that some of these genes may have acquired non-immune functions.

Our findings also shed light on the origin of ectodomains in the glycoproteins of filoviruses and reptarenaviruses. The presence of an ectodomain containing an immunosuppressive region in Ebola and Marburg viruses, and homology to the ectodomain of retroviruses, had been noted in ref. ^35^. Similarly, the glycoproteins of reptile arenaviruses (genus *Reptarenavirus*) were reported to be highly similar to the glycoproteins of filoviruses^36^. We could not detect the presence of the ectodomain in fish filoviruses (*Oblavirus*, *Striavirus*, *Thamnovirus*), or in other arenaviruses aside from *Reptarenavirus*. This patchy distribution suggests that the presence of the ectodomain is a derived character (apomorphy) in some filoviruses and *Reptarenavirus*, and not an ancestral trait for the families *Filoviridae* and *Arenaviridae*. These observations suggest a macroevolutionary scenario whereby retroviral ectodomains were captured twice by filoviruses and once by reptarenaviruses independently, over a timescale of hundreds of millions of years, pointing to the advantage gained by acquisition of the ectodomain in these viruses.

Our study demonstrated the capacity of cloud-based, highly parallelized approaches to harness vast amounts of sequence data, revealing multiple insights into the biology of viruses. We present evidence of endogenous chuvirus, paramyxovirus, plant-like virus (benyvirus), orthonairovirus and hepacivirus elements in vertebrate genomes. These discoveries open rich grounds to study the potential function of diverse non-retroviral EVEs on host biology. We foresee that with ever-increasing availability of genomic sequence data and the advance in computing power and algorithms, our knowledge of the genomic fossil record of viruses and their interactions over time will continue to increase.

## Methods

We used cloud computing on the Google Cloud Platform (https://cloud.google.com) to search for homology to a comprehensive set of protein sequences derived from viruses in the kingdoms *Shotokuvirae* (ssDNA and dsDNA viruses) and *Orthornavirae* (RdRp-containing RNA viruses) across all representative vertebrate genomes. We decided to focus our search on these two viral kingdoms, encompassing a diverse set of RNA and DNA virus lineages, since they have well-known EVE representatives, and we wished to explore whether a cloud-computing approach would allow us to discover EVEs from additional viral families in these well-characterized groups. These viral kingdoms also include animal pathogens, and their known diversity has expanded considerably in the past years. Hits to orthornaviruses and shotokuviruses were extracted and processed for taxonomic assignment into their respective viral groups (hits that did not return 50% reciprocal hits to viruses were considered ambiguous and not considered further). Hits showing high sequence similarity to known viruses or otherwise present in small contigs (<10,000 bp) without nearby host genes were considered exogenous viruses. Confirmed endogenous viral elements were then annotated, aligned and used in phylogenetic inference together with homologues from exogenous viruses. A more detailed description of the methods is described in the following sections.

### Selection of viral queries and sequence clustering

We downloaded 439,594 protein sequences from complete viral genomes available at NCBI Virus (https://www.ncbi.nlm.nih.gov/labs/virus/vssi/#/) in September 2022. The sequences were partitioned according to their viral family and clustered using MMSeqs2 (ref. ^55^). Clustering was performed using a minimum pairwise identity (–min_seq_id) of 65% at the amino acid level and the default cover (80%). Sequence centroids were extracted from each cluster and used as representative sequences for downstream analyses. This representative set contained 24,478 sequences.

### Elastic-BLAST searches on the Google Cloud Platform

Cloud searches for each viral family were conducted on the Google Cloud Platform (https://cloud.google.com) using the Elastic-BLAST algorithm^56^ in September 2022. Each search was performed with tblastn (tblastn-fast option) against the entire database of representative vertebrate genomes (ref_euk_rep_genomes, taxid: ‘7742’) and using an *e*-value of 1 × 10^−5^. The output was saved in tabular format (-outfmt ‘7’). The analysis returned 196,899 hits to the viral queries. We compared the cloud approach (using spot instances) to more traditional methods (local mmseqs2, diamond and tblastn-fast) with a benchmark search of all available bornavirus proteins (3,170 proteins, family *Bornaviridae*) against 34 representative primate genomes (taxid9443) (Supplementary Data file). Local searches were run on a single machine with 48 CPUs (Intel Xeon Gold 5220R @ 2.20 GHz × 48), 250.4 GiB RAM, 2.04 TB SSD (PC801 NVMe SK hynix), running Ubuntu 20.04.6 LTS OS. The mmseqs2 (tblastn mode) and diamond (blastx) search used all 48 threads, while the tblastn-fast search was run on 6 threads. We conducted every search with 5 replicates (*n* = 5). The fastest methods were mmseqs2 (100.83 ± 3.69 min), followed by elastic-blast (148.13 ± 30.22 min), diamond (167.48 ± 7.97 min) and lastly, tblastn-fast (1,398.02 ± 8.90 min) (Supplementary Fig. 6). Although mmseqs2 was the fastest method, hits are only provided for ORFs after prediction through the ‘extractorfs’ module. Therefore, we demonstrate that elastic-blast on the cloud is a performant method for interrogating large databases during EVE discovery workflows, which also need to detect sequences with degraded or absent ORFs. In addition, cloud searches (1) can be conducted with access to minimal computing resources (outsourced to the cloud), without the need to download large NCBI databases which are hosted natively on the cloud (decreased latency); (2) are scalable via parallelized workloads; and (3) reduce the local disk (storage) requirements since results can be stored directly in cloud buckets.

### Curation of non-redundant loci

Hits to host genomes were merged with bedtools2 (ref. ^57^) to reduce redundancy in the data set. Strictly overlapping hits and hits that were at a maximum distance of 200 nt (based on their genomic coordinates) were merged to give a single range in the host genome (-d 200). We thus obtained a set of 26,324 non-redundant genomic regions. We then downloaded the genomic sequences from the merged ranges in fasta format using efetch^58^. A list of the parent genome assemblies for these sequences is provided in Supplementary Data.

### DIAMOND reciprocal searches and taxonomic assignment

To assess the origin of the host sequences (whether viral or host), we downloaded and compiled the complete nr protein database with taxonomic information on the High-Performance Computing cluster at the University of Oxford. We then performed a reciprocal similarity search using the host sequences as queries and the nr database with DIAMOND blastx^59^, keeping only the top 25 hits. We obtained 558,589 reciprocal hits in total. Next, we used custom scripts written in Python 3 to parse the taxonomic labels obtained for each query sequence and assign them to the majority-rule viral family. Sequences were considered potentially viral if ≥50% of the reciprocal hits were to ‘Viruses’, and further confirmed via manual curation of each sequence. Parsing sequences based on the taxonomy of labels (before manual curation) had an estimated sensitivity (true positive rate) of 71.3%, a specificity (true negative rate) of 97.1%, precision of 98.2%, false discovery rate of 1.8% and an overall accuracy of 79.3% (Supplementary Table 1). Viral sequences falling on short contigs or with high similarity to known exogenous viruses (>99% identical) were considered exogenous viruses present in the assemblies (and not EVEs).

### Phylogenetic inference and structural predictions

We focused on elements that had not been described as EVEs in the literature for the phylogenetic and structural analyses. Predicted protein sequences for each locus were obtained and annotated manually using blastx/conserved domain search on the NCBI web server^60–62^, GeneWise on the EBI web server^63,64^ or HHpred on the Max Planck Institute’s web server^65,66^. Exogenous virus homologues were searched against the nr database using blastp online. Multiple sequence alignments were obtained using MAFFT^67^ or MACSE/translatorx^68,69^. Trees were estimated from amino acid data, except for nairoviruses which were based on a nucleotide alignment. We selected the best substitution models in Modeltest-NG^70^. Trees were estimated in RAxML-NG^71^ with 200 starting trees and up to 2,000 bootstraps (autoMRE{2000}) until convergence in MrBayes3 (ref. ^72^) (standard deviation of split frequencies <0.01) and in BEAST2 (ref. ^73^) (after inspecting the runs for good mixing, stationarity and effective sample sizes >200). For the inference of the timetree of ectodomains, we used orthology of the tarsier elements and estimated their ages by obtaining a corrected (TN93) genetic distance estimate between pairs of long terminal repeats (LTRs) in the same element, assuming a nucleotide substitution rate of 2.2 × 10^−3^ to 3 × 10^−3^ subst. per site per Myr^74,75^ (Supplementary Data). We used the estimated ages of these orthologues to calibrate internal nodes in the tree and used a prior distribution on the root of the tree, assuming that the retroelements present in cartilaginous fish/tetrapods codiverged with their gnathostome hosts (prior mean 462, prior 95% CI: 436–489 Ma). The posterior evolutionary rate of the ectodomains was estimated at 3.2 × 10^−9^ amino acid subst. per site per year (±4.4 × 10^−10^ aa subst. per site per year, Supplementary Fig. 5). This is consistent with the higher neutral evolutionary rates reported for immunoglobulin kappa (3.7 × 10^−9^ aa subst. per site per year) and gamma C chains (3.1 × 10^−9^ aa subst. per site per year), and the complement C3a anaphylatoxin (2.7 × 10^−9^ aa subs. per site per year)^76^. It is also consistent with the time dependency of viral evolutionary rates, which tend to converge on the host rate over geological timescales^77^. These observations indicate that the timescale of evolution was calibrated properly; misspecified priors would have resulted in a substantial departure from the time-dependent and neutral expectations.

Cophylogenetic analysis for benyviruses was performed and plotted in Rtapas using the maximum incongruence algorithm^78^. We predicted select paramyxovirus and nairovirus protein structures using AlphaFold2 (ref. ^79^) as implemented in ColabFold^80^. We used amber relaxation on the top-ranked structure and either 24 or 48 recycles. Network analysis of chuvirus capsid proteins was performed using CLANS 2.0 (refs. ^81,82^), with a *P* < 1 × 10^−15^; the choice to perform a network analysis was due to the low support values obtained for tree topologies using both maximum-likelihood and Bayesian methods.

Placement of the Etruscan shrew EVE as a member of the genus *Orthonairovirus* was done following a phylogenetic criterion. Following the ICTV taxonomy, currently recognized orthonairoviruses include: Nayun tick virus, Erve virus, Thiafora virus, Wufeng Crocidura attenuata orthonairovirus 1, Hazara virus, Tofla virus, Meihua Mountain virus, Nairobi sheep disease virus, Meram virus and Crimean–Congo hemorrhagic fever virus (CCHFV)^83^. Since the Etruscan shrew EVE was firmly placed within this clade, it can be confidently assigned to the genus *Orthonairovirus*. Similarly, the EVEs discovered in the ticks *Dermacentor silvarum*, *D. andersoni* and *Rhipicephalus sanguineus* can also be assigned to the genus *Orthonairovirus*.

### Systematic search of orthology

Orthology was systematically searched on the basis of a code we developed (Orthology.py), which takes a set of sequence accessions and genomic coordinates, and gives a list of potential orthogroups on the basis of pairwise alignments of the host flanking sequences. First, the upstream and downstream flanks of each sequence were downloaded (size set by ‘–flank_size’ parameter), followed by an all-against-all blastn search (*e*-value = 1 × 10^−5^). A data frame was then populated with the outcomes of each pairwise comparison, with 1s in each cell where an alignment equal to or over the coverage threshold (‘–coverage_threshold’) was found, and 0s when there was no alignment meeting this criterion. A reduced data frame was then built where each 2 × 2 submatrix was interrogated as to whether there was an alignment found between the upstream/downstream flanks of two sequences (represented as 1 if there is, 0 if none). This reduced data frame defined an adjacency matrix that could be converted into an undirected graph where each sequence was represented by a node, and nodes where an alignment was detected were joined by edges. In this way, clusters of connected nodes (‘components’, a connected subgraph not part of any larger subgraph) could be extracted; these in turn, represented potential orthogroups. We used this approach to examine orthology of all the predicted EVEs using a flank size of 2 kb, a coverage threshold of 70% and at least 1 significant alignment found during submatrix interrogation (the orthogroup Circo_Rep_14 was further confirmed by using stricter criteria given its initial size: flank size = 30 kb, 95% coverage and 2 or more alignments in the interrogated submatrices). Each potential orthogroup was further validated manually by alignment of each EVE locus in its orthogroup with ±5 kb flanks and confirmed by visual inspection of the alignments.

The orthologues shown in Fig. 1c were identified on the basis of the syntenic arrangement of most-proximal genes. Briefly, chuvirus EVEs were queried against the representative RefSeq genomes on the NCBI, and hits with good cover and high percentage identity were shortlisted. We then checked the genomic context of the alignments in the Genome Data Viewer and visualized the annotations of nearby genes. Three EVE pairs are shown whose orthology was confirmed via this method.

### Open reading frame prediction and estimation of d*N*/d*S*

We predicted ORFs in the orthogroups and kept ORFs that encoded products of at least 100 amino acids in 2 members. We curated the set of predicted ORFs to confirm that they encoded the EVE of interest. Codon-phased, multiple sequence alignments were then estimated for each orthologous set of ORFs using translatorx^69^. We then tested for selection by fitting a neutral branch model (d*N*/d*S* fixed to 1), a single estimated omega (single d*N*/d*S* estimated for all branches) and a free ratio model (d*N*/d*S* free to vary across branches) using codeml on PAML (v.4.10.6)^84^. Orthogroups with only 2 sequences were analysed in the ‘pairwise’ mode (runmode = −2), while groups with 3 or more sequences were analysed in ‘user tree’ mode (runmode = 0). Trees were inferred in RAxML v.1.2.0 after selecting the best substitution model in Modeltest-NG. The models were compared to each other using likelihood-ratio tests. The test statistic was calculated as twice the difference in the log-likelihoods of the models (lowest minus highest), together with the difference in the degrees of freedom of the models. We searched for the *P* value of the comparison under the Chi-squared distribution using the function pchisq() with ‘lower.tail = FALSE’ in R and only accepted an alternative hypothesis when *P* < 0.05.

## Statistics and reproducibility

No statistical method was used to predetermine sample size. All sequence data described were included in their respective analyses. The investigators were not blinded during data collection or subsequent analyses.

### Reporting summary

Further information on research design is available in the Nature Portfolio Reporting Summary linked to this article.

## Supplementary information

Supplementary Information Supplementary Figs. 1–6, and Tables 1 and 2.

Reporting Summary

Supplementary Data Supplementary data, pages 1–25.

## Source data

Source Data Fig. 1 RdRp tree run files (Fig. 1a), CLANS analysis data (Fig. 1b) and xlsx file with genomic annotations of sequences (Fig. 1c).

Source Data Fig. 2 RdRp tree files (Fig. 2a), *Astyanax mexicanus* structural modelling files (Fig. 2b) and NP tree files (Fig. 2c).

Source Data Fig. 3 eEF1a and RdRp tree files, host/virus trees, host–virus association matrix and R script used to conduct the cophylogenetic analysis.

Source Data Fig. 4 NP tree files (Fig. 4a), and structural modelling files for the *S. etruscus* EVE, *Ixodes scapularis* EVE and South Bay virus nucleoprotein (Fig. 4b).

Source Data Fig. 5 Sequence data and alignments used in the analysis of the endogenous hepacivirus-like EVE in murine rodent genomes.

Source Data Fig. 6 Timetree files used in the analysis of viral ectodomains.

Source Data Supplementary Fig. 1 R script used to plot hit distributions (untransformed values).

Source Data Supplementary Fig. 2 R script used to plot hit distributions (log-transformed).

Source Data Supplementary Fig. 3 RdRp tree files used in the analysis of the benyvirus-like EVEs in vertebrate genomes.

Source Data Supplementary Fig. 4 Ectodomain tree files used to choose the ingroup for the time-calibrated evolutionary analysis.

Source Data Supplementary Fig. 5 Mean evolutionary rate burn-in file and R script used to plot the rate distribution.

Source Data Supplementary Fig. 6 File of bornavirus proteins used to query the 34 primate genomes, together with the duration csv file and the R script used to plot the figure.

Source Data Supplementary Table 1 Manually classified samples of predicted host and virus sequences used to calculate the proportions shown in Supplementary Table 1.

Source Data Supplementary Table 2 csv file with the duration of each run for each algorithm.

## Data Availability

All data and code supporting this work are available on the Open Science Framework server at https://osf.io/7rqa2 (ref. ^85^). Thirty-three genome assemblies screened in this study were released by the Vertebrate Genomes Project and analysed in line with the VGP embargo policy (only 5 EVEs described for 2 species still under embargo: Asian elephant and Ptarmigan). In this study, the use of the Asian elephant and ptarmigan genomes was permitted under exception of the VGP embargo policy since 5 loci are described in 2 species. The VGP embargo policy exceptions are for analyses of either a single locus, a single gene family in a species, a maximum of 5 gene loci across multiple species, or for use as a reference for mapping reads from independent studies. For more information, see https://genome10k.ucsc.edu/data-use-policies. Accession numbers, BioProject and BioSample IDs of the assemblies analysed in this study can be found in Supplementary Data. All data and metadata for these sequence records are publicly available and can be accessed via the NCBI (US National Centre for Biotechnology Information) at www.ncbi.nlm.nih.gov. Source data are provided with this paper.

## Code availability

The custom code developed for this study is also available in OSF (https://osf.io/7rqa2) which has been linked to the main GitHub repository^85^.
