# Supplementary material for: Deep mining reveals the diversity of endogenous viral elements in vertebrate genomes
Source: Nat Microbiol. 2024 Oct 22;9(11):3013–24. doi: 10.1038/s41564-024-01825-4 (PMC11521997; doi:10.1038/s41564-024-01825-4)
Supplement: Supplementary file 1 — Supplementary Figs. 1–6, and Tables 1 and 2. [file 41564_2024_1825_MOESM1_ESM.pdf]

nature microbiology

Supplementary information https://doi.org/10.1038/s41564-024-01825-4

Deep mining reveals the diversity of endogenous viral elements in vertebrate genomes

In the format provided by the authors and unedited

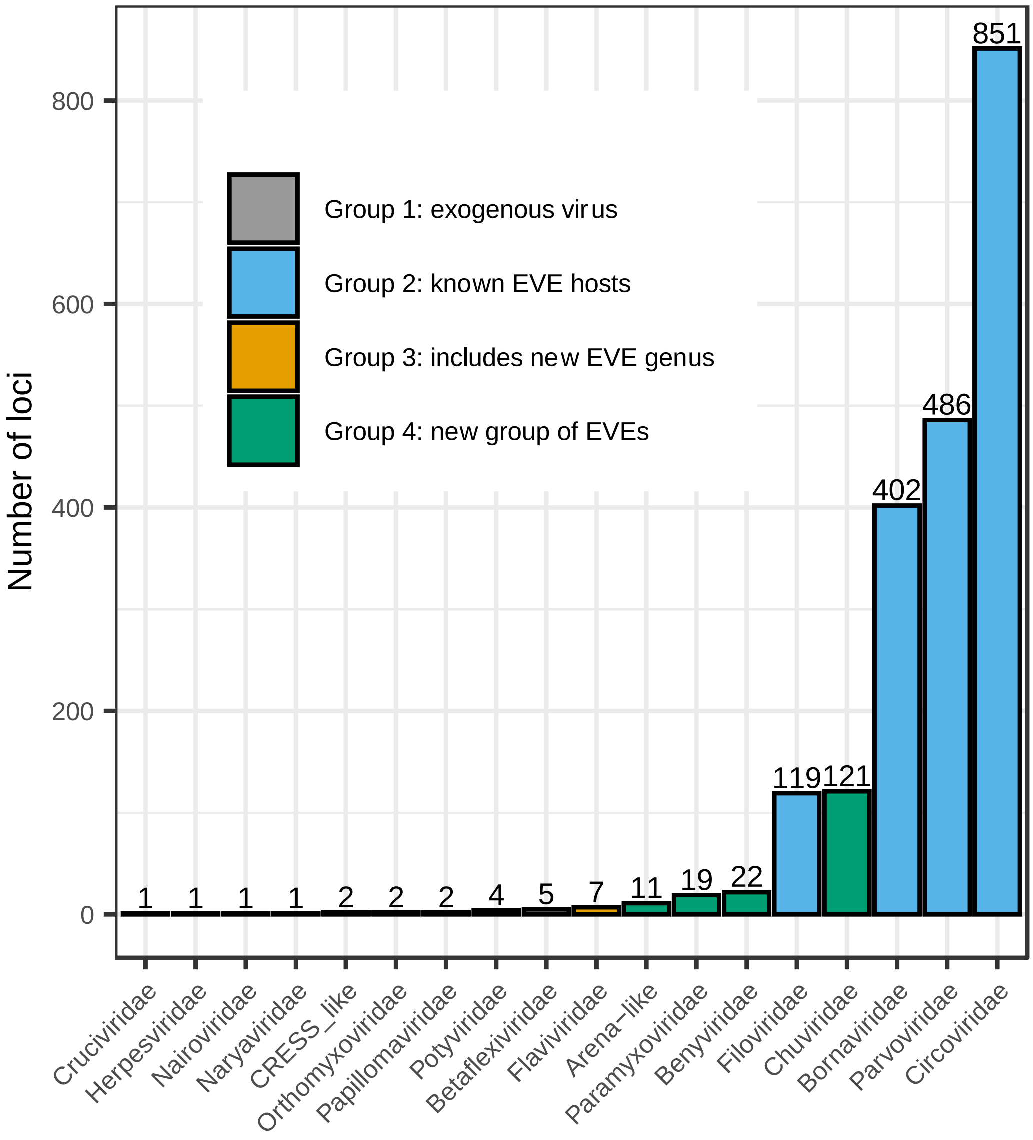

Supplementary figure 1. Distribution of non-redundant hits to viruses in representative vertebrate genomes (absolute numbers). In total 2,057 sequences were detected. Circovirus, parvovirus and bornavirus EVEs (1,739), account for 85.2% of all EVEs found (2,040). Sequences were divided into 4 groups depending on whether they are likely exogenous viruses (Group 1, grey), EVEs in known hosts (Group 2, blue), EVEs that include a new genus (Group 3, orange) or a new group of EVEs (Group 4, green). The “Arena-like” group refers to ectodomains with high similarity to reptarenavirus ectodomains, despite being embedded within retrovirus-like elements.

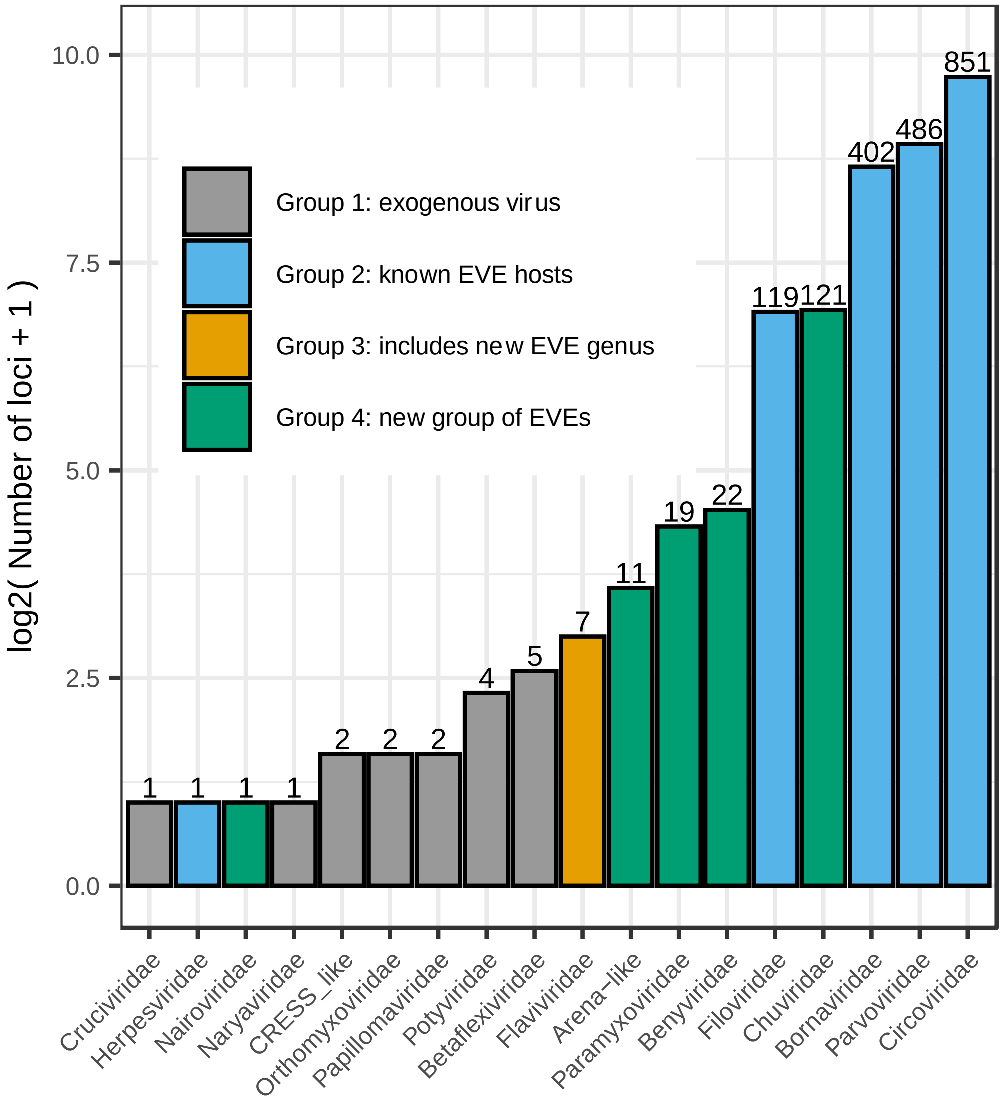

Supplementary figure 2. Distribution of non-redundant hits to viruses in representative vertebrate genomes (log2 scale). We used a log2-scale to facilitate comparisons of abundance. In total 2,057 sequences were detected. Circovirus, parvovirus and bornavirus EVEs (1,739), account for 85.2% of all EVEs found (2,040). Sequences were divided into 4 groups depending on whether they are likely exogenous viruses (Group 1, grey), EVEs in known hosts (Group 2, blue), EVEs that include a new genus (Group 3, orange) or a new group of EVEs (Group 4, green). The “Arena-like” group refers to ectodomains with high similarity to reptarenavirus ectodomains, despite being embedded within retrovirus-like elements.

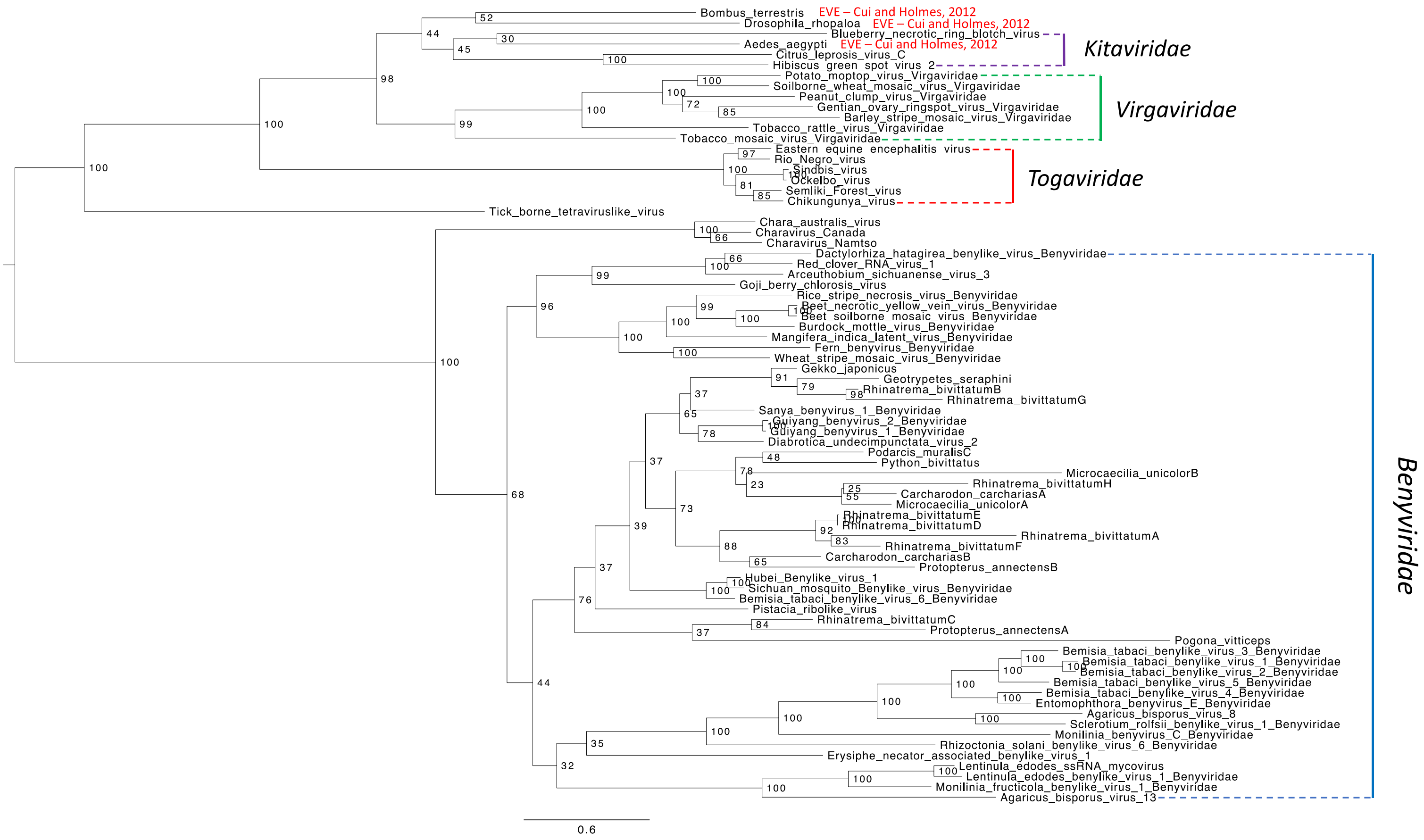

Supplementary figure 3. Midpoint-rooted maximum-likelihood tree of the RNA-dependent RNA polymerase of benyviruses, togaviruses, virgaviruses and kitaviruses, together with endogenous elements. Exogenous benyviruses and the vertebrate beny-like elements form a highly supported monophyletic group (100% bootstrap support) with charaviruses (which infect the charophyte alga *Chara*). Previously described plant virus-like EVEs in insect genomes (Cui and Holmes, 2012), fall closer to kitaviruses and virgaviruses. Tree inferred in RAxML-NG using the LG+FC+I+G4 model and 950 bootstrap replicates (until convergence).

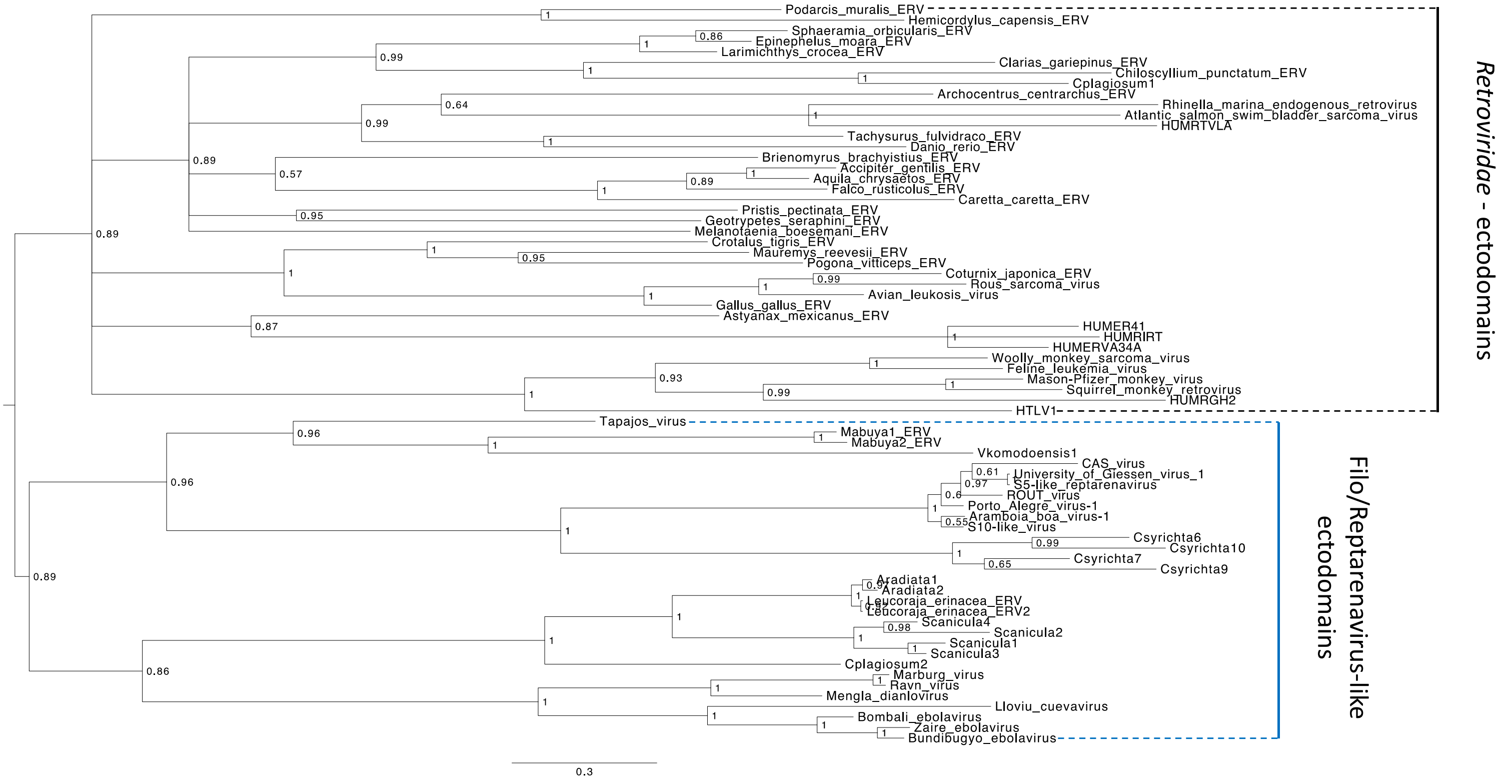

Supplementary figure 4. Midpoint-rooted Bayesian tree of ectodomains found in retroviruses, filoviruses, reptarenaviruses and endogenous elements in vertebrate genomes. The endogenous ectodomains found in the genomes of tarsiers (Csyrichta) and cartilaginous fish (Aradiata, Leucoraja, Scanicula, Cplagiosum) were placed in a clade (posterior probability = 0.89) together with the ectodomains of exogenous reptarenaviruses and filoviruses. Other retroviral sequences were placed outside this group. Tree inferred in MrBayes3 using the Vt+G4 model, 10 million generations and a 25% relative burn-in.

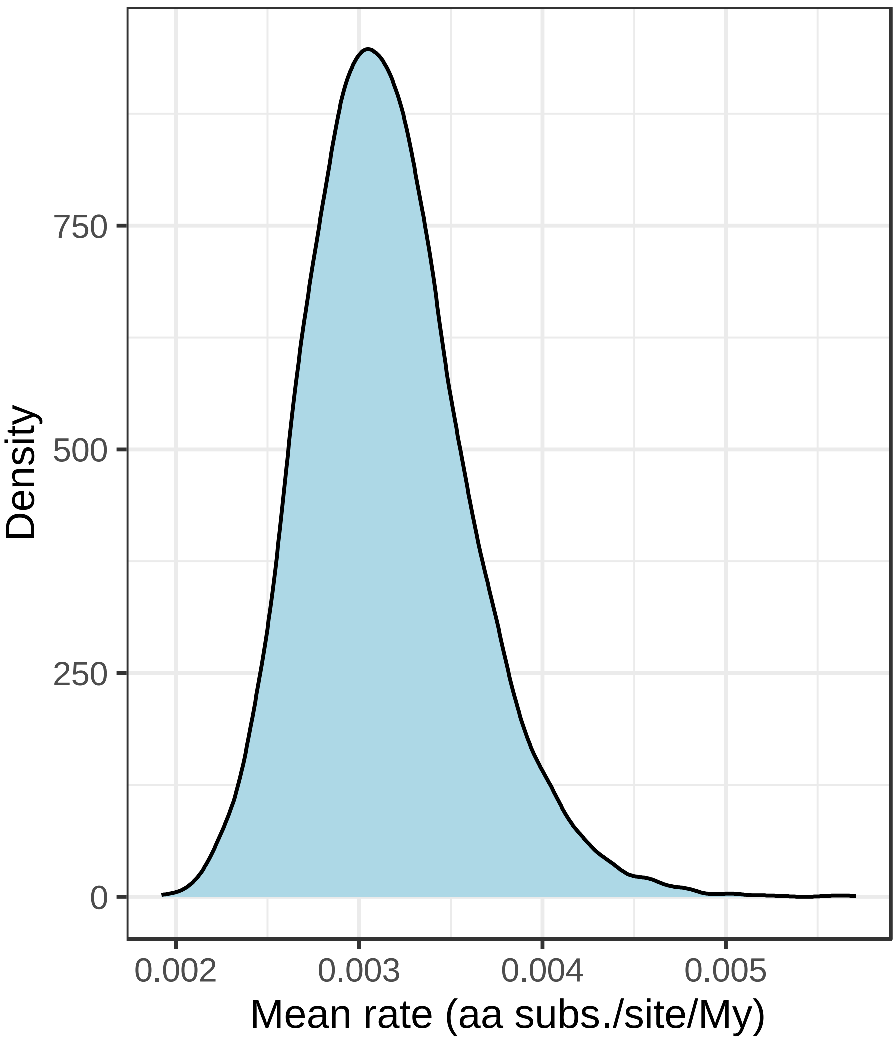

Supplementary figure 5. Distribution of mean evolutionary rates in the BEAST2 analysis. The mean evolutionary rate of the ectodomains was estimated at $3.2 \cdot 10^{-3}$ amino acid substitutions per site per million years (aa subs./site/My), with a standard deviation of $4.4 \cdot 10^{-4}$ (aa subs./site/My). Coefficient of variation = 0.14 (14%). Relative burn-in = 25%.

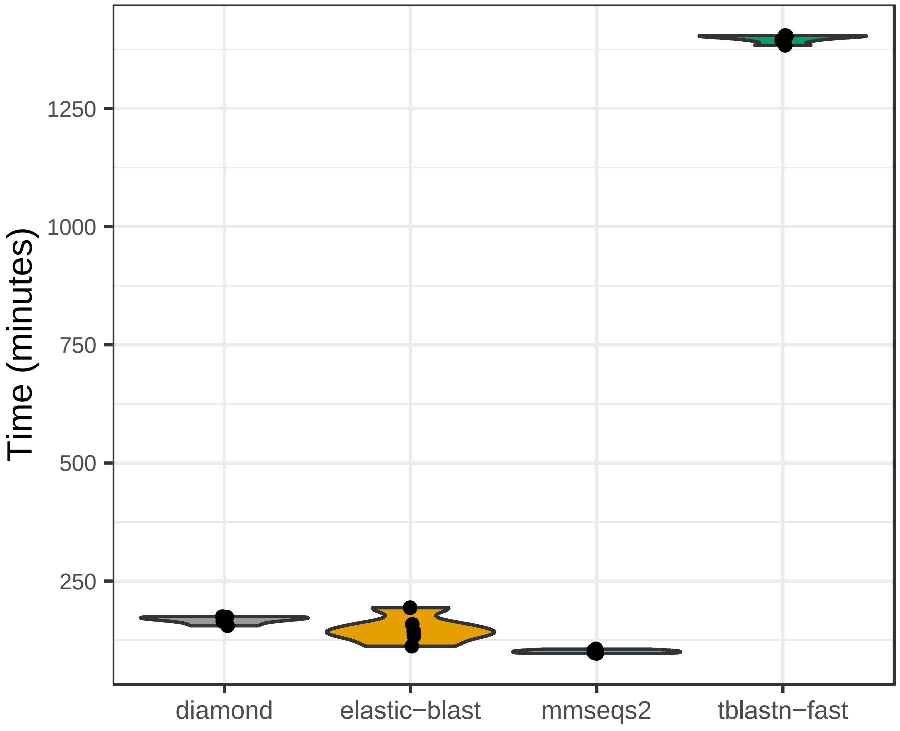

Supplementary figure 6. Runtimes of the elastic-blast sequence-similarity search method, compared to local approaches (mmseqs2, diamond and tblastn-fast). Each method was run with 5 replicates (n = 5), using a benchmark dataset of all proteins for the family *Bornaviridae* (3,170 proteins) and 34 representative primate genomes. The table inset shows the methods ranked by speed, with mean and standard deviation shown in minutes (min). Mmseqs2 was the fastest method (~1.68 hours), followed by elastic-blast (~2.46 hours), diamond (~2.79 hours) and local tblastn-fast was the slowest (~23.3 hours). However, the mmseqs2 results are restricted to hits which match open reading frames predicted by the 'extractorfs' module.

Supplementary Table 1. Confusion matrix for the classification of sequences based on the presence of ≥50% labels to "Viruses". The values have been normalised on the predicted class (column-wise normalisation). The estimates were obtained by partitioning the set of reciprocal hits into predicted viral (positive) and predicted non-viral (negative) groups. We took 10 random samples of 50 sequences (500 sequences) for each group, and then manually curated each sequence for assignment into an actual class (viruses: positive, host: negative). The mean estimate is shown, plus minus the standard deviation.

**Class**	Predicted Positive (%)	Predicted Negative (%)
Actual Positive	98.2 ± 1.5	39.6 ± 8.7
Actual Negative	1.8 ± 1.5	60.4 ± 8.7

Sensitivity (True positive rate, TPR) = 71.3%
Specificity (True negative rate, TNR) = 97.1%
Accuracy = 79.3%
Precision (Positive Predictive Value, PPR) = 98.2%
False discovery rate (FDR) = 1.8%

Supplementary Table 2. Runtimes of different similarity search algorithms benchmarked on 3,170 bornavirus proteins screened against 34 representative primate genomes (taxid9443). Each method was tested with 5 replicates (n = 5). Further details are explained in the legend of Supplementary figure 6.

Rank	Algorithm	Mean (min)	Standard deviation (min)
1	mmseqs2	100.83	3.69
2	elastic-blast	148.13	30.22
3	diamond	167.48	7.97
4	tblastn-fast	1398.02	8.90
