# Supplementary material for: Deep mining reveals the diversity of endogenous viral elements in vertebrate genomes
Source: Nat Microbiol. 2024 Oct 22;9(11):3013–24. doi: 10.1038/s41564-024-01825-4 (PMC11521997; doi:10.1038/s41564-024-01825-4)
Supplement: Supplementary file 2 — Reporting Summary [file 41564_2024_1825_MOESM2_ESM.pdf]

nature portfolio

Corresponding author(s): Aris Katzourakis

Last updated by author(s): Jul 4, 2024

Reporting Summary

Nature Portfolio wishes to improve the reproducibility of the work that we publish. This form provides structure for consistency and transparency in reporting. For further information on Nature Portfolio policies, see our Editorial Policies and the Editorial Policy Checklist.

Statistics

For all statistical analyses, confirm that the following items are present in the figure legend, table legend, main text, or Methods section.

n/a	Confirmed	
☐	☒	The exact sample size (*n*) for each experimental group/condition, given as a discrete number and unit of measurement
☐	☒	A statement on whether measurements were taken from distinct samples or whether the same sample was measured repeatedly
☐	☒	The statistical test(s) used AND whether they are one- or two-sided<br>*Only common tests should be described solely by name; describe more complex techniques in the Methods section.*
☒	☐	A description of all covariates tested
☐	☒	A description of any assumptions or corrections, such as tests of normality and adjustment for multiple comparisons
☐	☒	A full description of the statistical parameters including central tendency (e.g. means) or other basic estimates (e.g. regression coefficient) AND variation (e.g. standard deviation) or associated estimates of uncertainty (e.g. confidence intervals)
☐	☒	For null hypothesis testing, the test statistic (e.g. *F*, *t*, *r*) with confidence intervals, effect sizes, degrees of freedom and *P* value noted<br>*Give P values as exact values whenever suitable.*
☐	☒	For Bayesian analysis, information on the choice of priors and Markov chain Monte Carlo settings
☒	☐	For hierarchical and complex designs, identification of the appropriate level for tests and full reporting of outcomes
☒	☐	Estimates of effect sizes (e.g. Cohen's *d*, Pearson's *r*), indicating how they were calculated

Our web collection on statistics for biologists contains articles on many of the points above.

Software and code

Policy information about availability of computer code

Data collection	mmseqs2 version 3e436173321a2d0365a08bfb3b281108ed9ad414<br>elastic-blast version 0.2.6
Data analysis	ncbi-blast version 2.14.0+<br>diamond version 2.1.6<br>bedtools version 2.27.1<br>modeltest-ng version 0.2.0<br>raxml-ng version 1.2.0<br>PAML version 4.10.6<br>CLANS 2.0<br>MrBayes version 3.2.7a<br>BEAST2 version 2.7.5<br>MAFFTversion 7.490<br>MACSE version 2.06<br>translatorx version 1.1<br>R version 4.3.2<br>Rtapas version 1.1<br>Python version 3.8.10<br>ColabFold: https://colab.research.google.com/github/sokrypton/ColabFold/blob/main/AlphaFold2.ipynb<br>NCBI BLAST: https://blast.ncbi.nlm.nih.gov/Blast.cgi<br>Genome Data Viewer: https://www.ncbi.nlm.nih.gov/gdv/

GeneWise: https://www.ebi.ac.uk/jdispatcher/psa/genewise
HHpred: https://toolkit.tuebingen.mpg.de/tools/hhpred
TimeTree: http://www.timetree.org/
GitHub repository: https://github.com/josegabrielnb/deep-mining

For manuscripts utilizing custom algorithms or software that are central to the research but not yet described in published literature, software must be made available to editors and reviewers. We strongly encourage code deposition in a community repository (e.g. GitHub). See the Nature Portfolio guidelines for submitting code & software for further information.

Data

Policy information about availability of data

All manuscripts must include a data availability statement. This statement should provide the following information, where applicable:

- Accession codes, unique identifiers, or web links for publicly available datasets
- A description of any restrictions on data availability
- For clinical datasets or third party data, please ensure that the statement adheres to our policy

All data and code supporting this work are available at the Open Science Framework and GitHub repositories: https://osf.io/7rqa2 and https://github.com/josegabrielnb/deep-mining.

Databases:

NCBI: https://www.ncbi.nlm.nih.gov/

Research involving human participants, their data, or biological material

Policy information about studies with human participants or human data. See also policy information about sex, gender (identity/presentation), and sexual orientation and race, ethnicity and racism.

Reporting on sex and gender	N/A.
Reporting on race, ethnicity, or other socially relevant groupings	N/A.
Population characteristics	N/A.
Recruitment	N/A.
Ethics oversight	N/A.

Note that full information on the approval of the study protocol must also be provided in the manuscript.

Field-specific reporting

Please select the one below that is the best fit for your research. If you are not sure, read the appropriate sections before making your selection.

☐ Life sciences ☐ Behavioural & social sciences ☒ Ecological, evolutionary & environmental sciences

For a reference copy of the document with all sections, see nature.com/documents/nr-reporting-summary-flat.pdf

Ecological, evolutionary & environmental sciences study design

All studies must disclose on these points even when the disclosure is negative.

Study description	Genomic analysis of endogenous viral elements (EVEs) of all vertebrate representative refseq genomes on the NCBI.
Research sample	Vertebrate genomes hosted in the ref_euk_rep_genomes database (taxid7742).
Sampling strategy	All available genomes in the above mentioned database were queried.
Data collection	The data was curated with the workflow described in the method section of the study. Briefly, all hits with an e-value < 1e-5 were downloaded, merged, assigned to a preliminary class (virus/non-virus) after a reciprocal diamond search (e-value 1e-5) and manually curated. Homologous sequences were then aligned and phylogenetic inference was performed.
Timing and spatial scale	ref_euk_rep_genomes database accessed in September, 2022.
Data exclusions	We included a set of 2040 sequences which returned >= 50% labels to viruses in the reciprocal search. These sequences were then

Data exclusions	manually curated for validation. The performance metrics for the parsing step of this strategy are described in the manuscript and in the confusion matrix presented in Supplementary Table 1.
Reproducibility	Given that accession numbers, coordinates, sequence sets and analyses are described in detail, our findings can be easily reproduced. We also make available the raw and curated data sets that we used for downstream analyses (OSF repository).
Randomization	All the EVEs detected in our pipeline were included in the study.
Blinding	Blinding was not necessary for the purpose of our study since we were not considering the effects of treatments on experimental/ control groups.

Did the study involve field work? ☐ Yes ☒ No

Reporting for specific materials, systems and methods

We require information from authors about some types of materials, experimental systems and methods used in many studies. Here, indicate whether each material, system or method listed is relevant to your study. If you are not sure if a list item applies to your research, read the appropriate section before selecting a response.

Materials & experimental systems

n/a	Involved in the study	
☒	☐	Antibodies
☒	☐	Eukaryotic cell lines
☒	☐	Palaeontology and archaeology
☒	☐	Animals and other organisms
☒	☐	Clinical data
☒	☐	Dual use research of concern
☒	☐	Plants

Methods

n/a	Involved in the study	
☒	☐	ChIP-seq
☒	☐	Flow cytometry
☒	☐	MRI-based neuroimaging

Plants

Seed stocks	N/A.
Novel plant genotypes	N/A.
Authentication	N/A.
