# Supplementary figures and images for: Deep mining reveals the diversity of endogenous viral elements in vertebrate genomes
Source: Nat Microbiol. 2024 Oct 22;9(11):3013–24. doi: 10.1038/s41564-024-01825-4 (PMC11521997; doi:10.1038/s41564-024-01825-4)

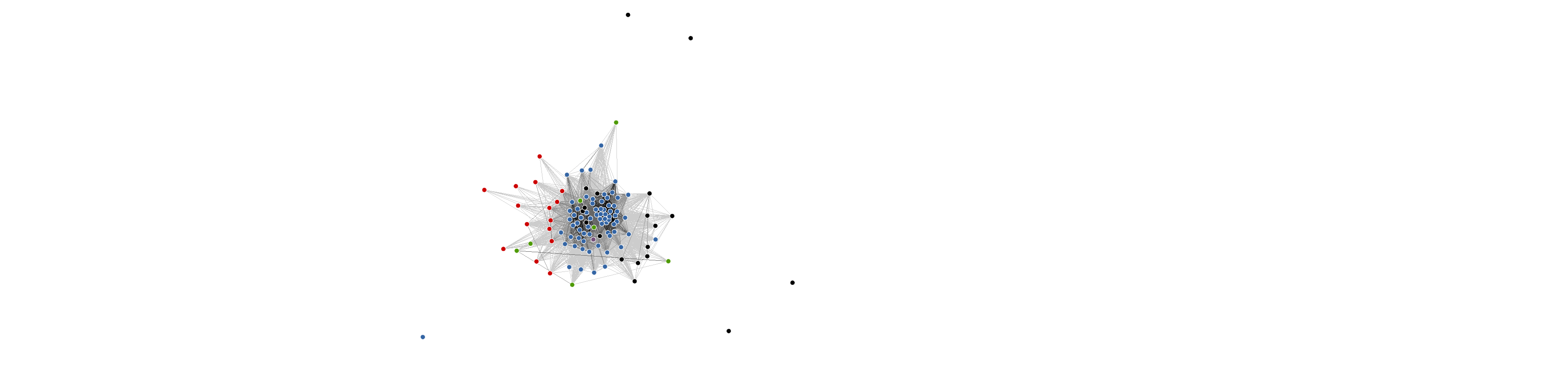

Supplement: Supplementary file 4 — RdRp tree run files (Fig. 1a), CLANS analysis data (Fig. 1b) and xlsx file with genomic annotations of sequences (Fig. 1c). [file 41564_2024_1825_MOESM4_ESM.zip › Source_Data_Figure_1/clans/clans.tiff]

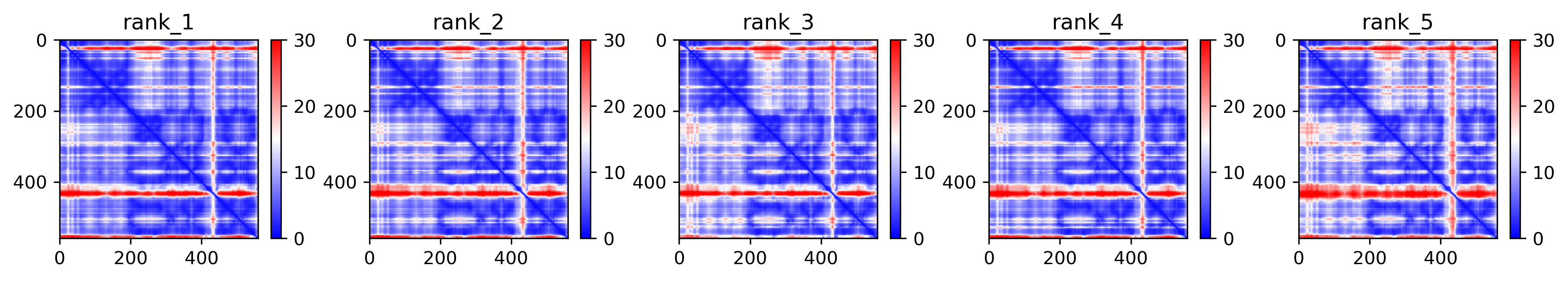

Supplement: Supplementary file 5 — RdRp tree files (Fig. 2a), Astyanax mexicanus structural modelling files (Fig. 2b) and NP tree files (Fig. 2c). [file 41564_2024_1825_MOESM5_ESM.zip › Source_Data_Figure_2/Astyanax_mexicanus559_RNApol_24recycles_amber_9cab3/Astyanax_mexicanus559_RNApol_24recycles_amber_9cab3_pae.png]

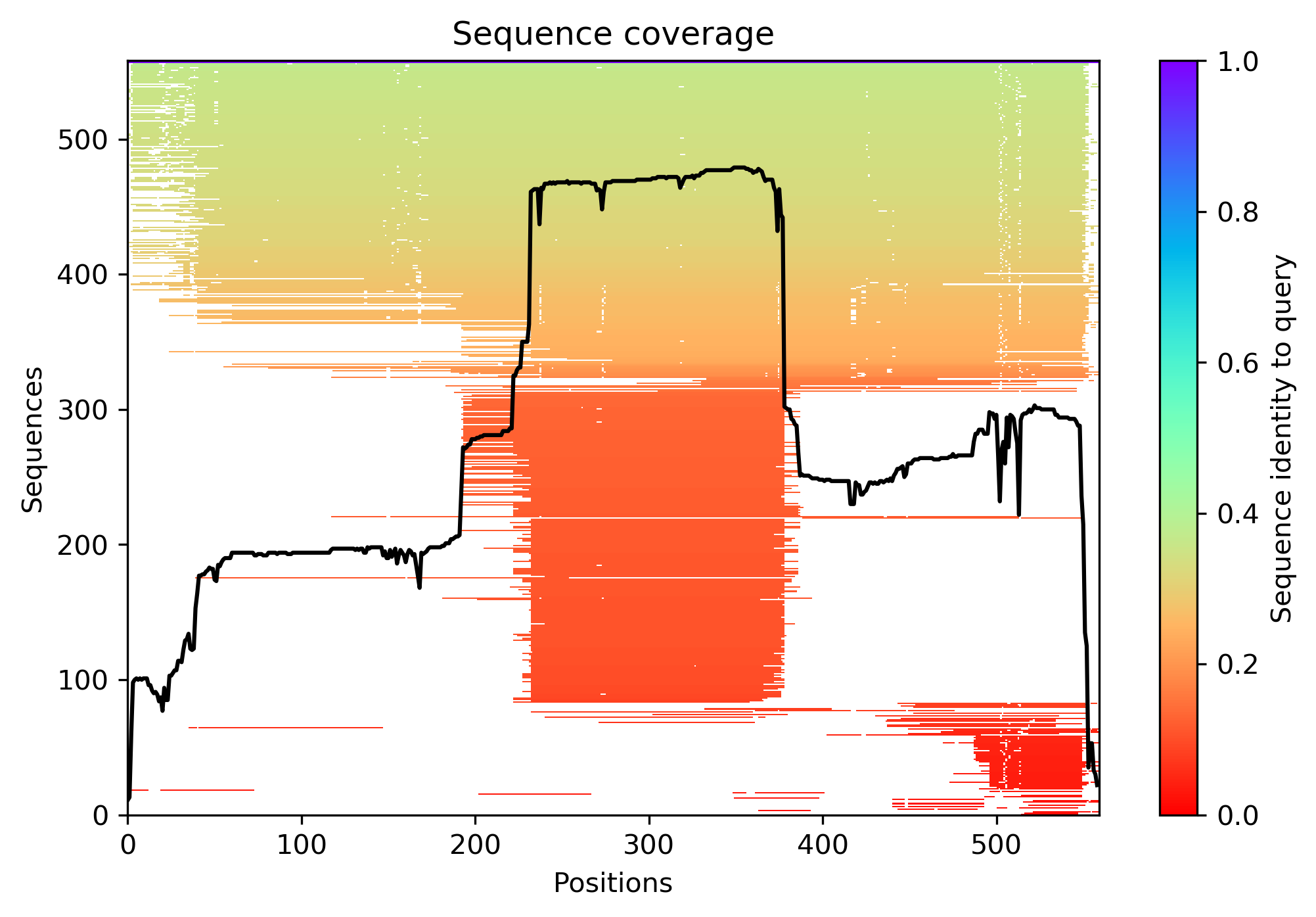

Supplement: Supplementary file 5 — RdRp tree files (Fig. 2a), Astyanax mexicanus structural modelling files (Fig. 2b) and NP tree files (Fig. 2c). [file 41564_2024_1825_MOESM5_ESM.zip › Source_Data_Figure_2/Astyanax_mexicanus559_RNApol_24recycles_amber_9cab3/Astyanax_mexicanus559_RNApol_24recycles_amber_9cab3_coverage.png]

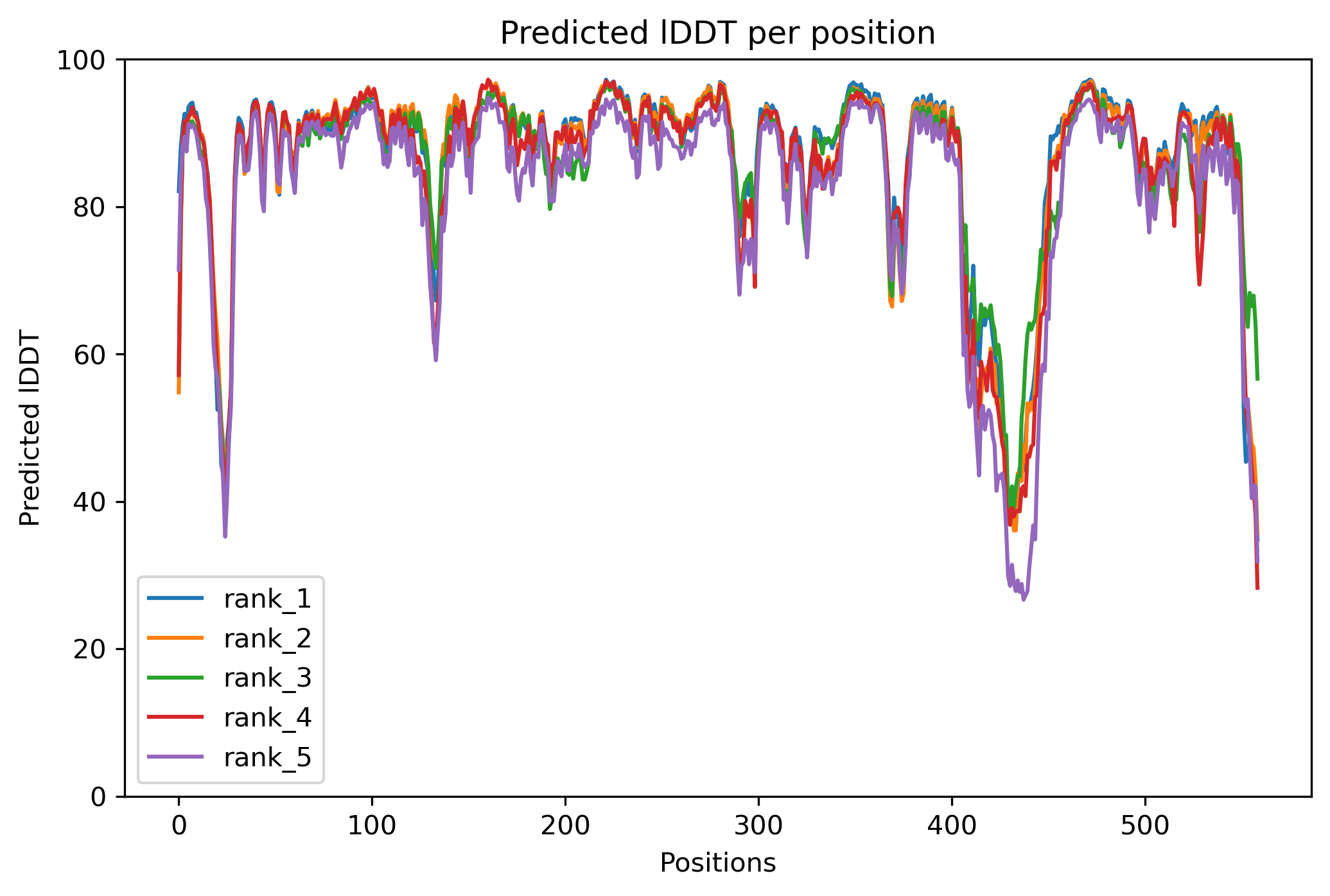

Supplement: Supplementary file 5 — RdRp tree files (Fig. 2a), Astyanax mexicanus structural modelling files (Fig. 2b) and NP tree files (Fig. 2c). [file 41564_2024_1825_MOESM5_ESM.zip › Source_Data_Figure_2/Astyanax_mexicanus559_RNApol_24recycles_amber_9cab3/Astyanax_mexicanus559_RNApol_24recycles_amber_9cab3_plddt.png]

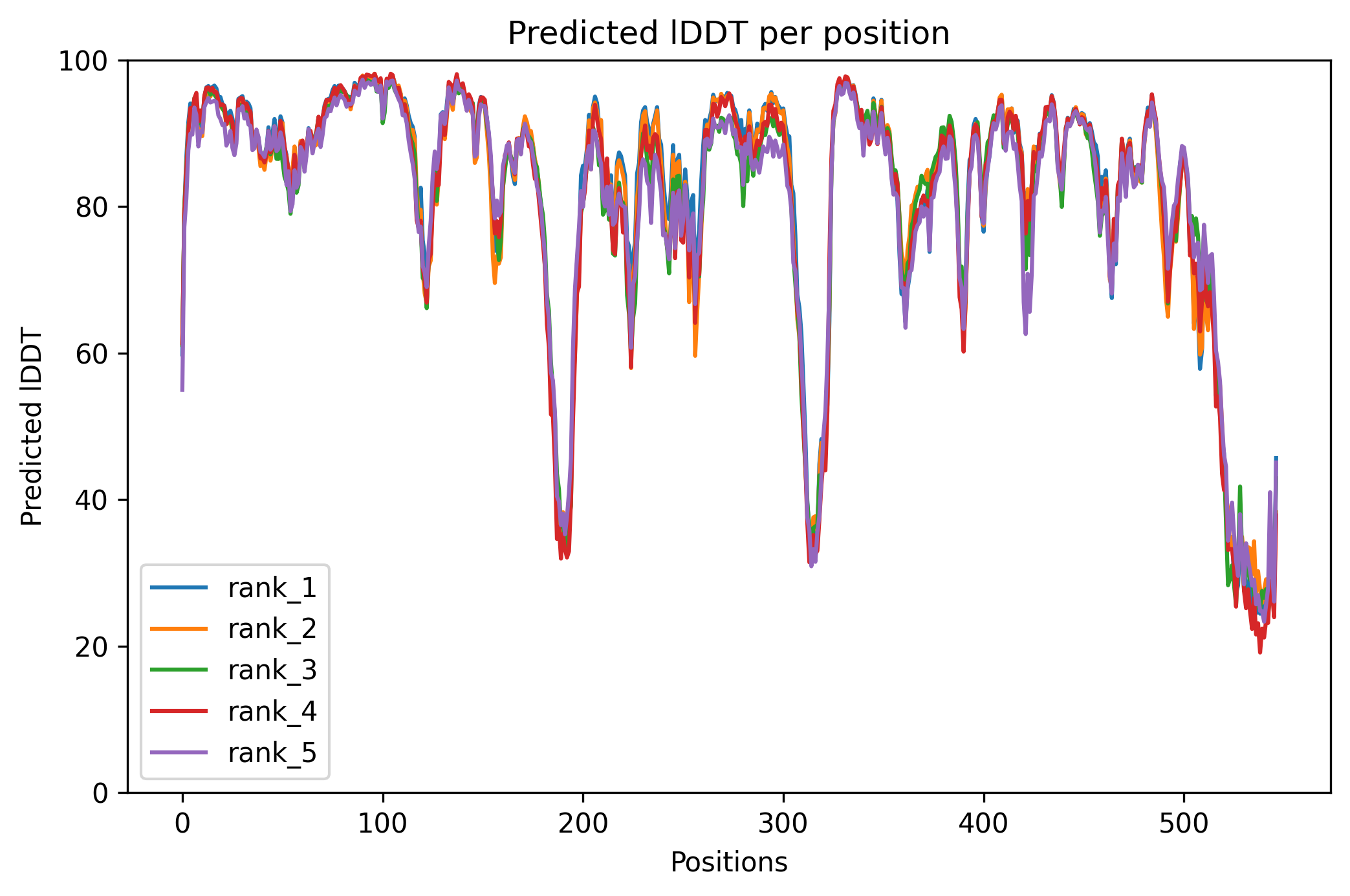

Supplement: Supplementary file 7 — NP tree files (Fig. 4a), and structural modelling files for the S. etruscus EVE, Ixodes scapularis EVE and South Bay virus nucleoprotein (Fig. 4b). [file 41564_2024_1825_MOESM7_ESM.zip › Source_Data_Figure_4/Ixodes_scapularis547_24recycles_amber_9dc3a/Ixodes_scapularis547_24recycles_amber_9dc3a_plddt.png]

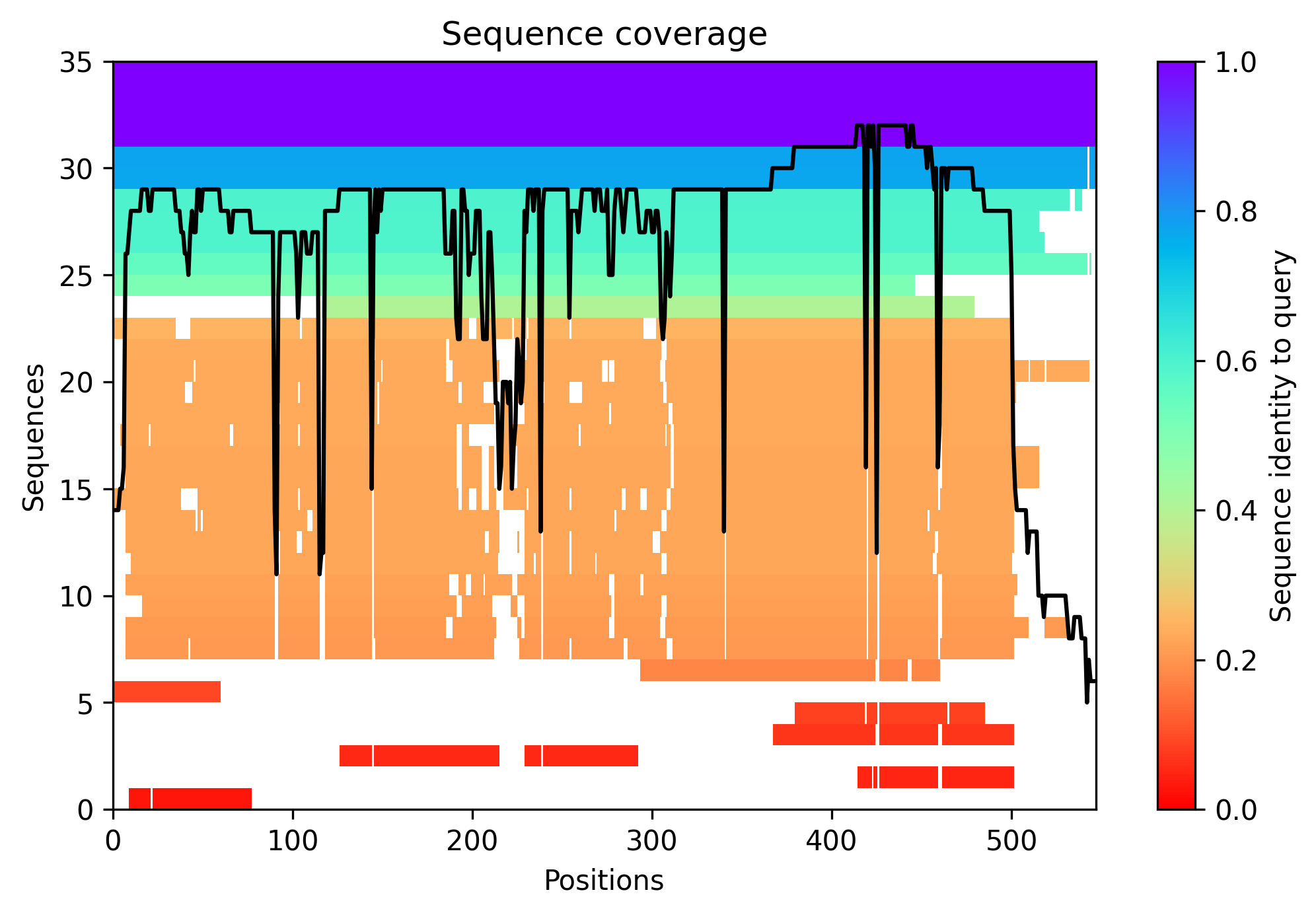

Supplement: Supplementary file 7 — NP tree files (Fig. 4a), and structural modelling files for the S. etruscus EVE, Ixodes scapularis EVE and South Bay virus nucleoprotein (Fig. 4b). [file 41564_2024_1825_MOESM7_ESM.zip › Source_Data_Figure_4/Ixodes_scapularis547_24recycles_amber_9dc3a/Ixodes_scapularis547_24recycles_amber_9dc3a_coverage.png]

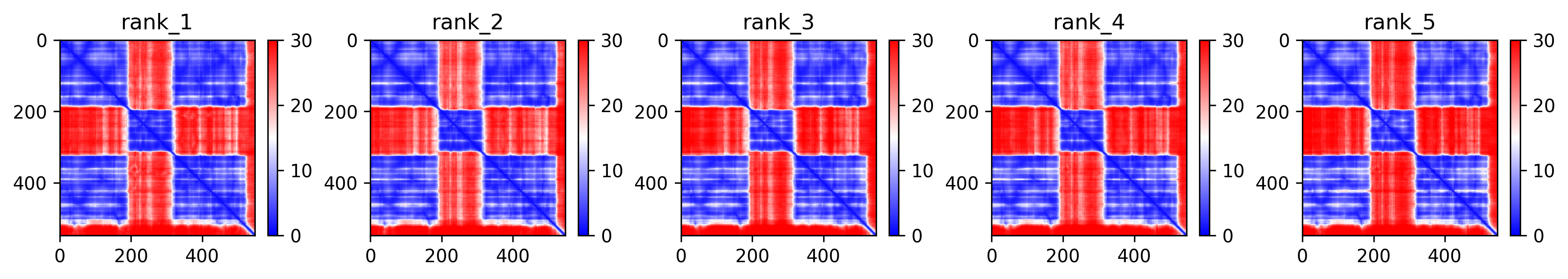

Supplement: Supplementary file 7 — NP tree files (Fig. 4a), and structural modelling files for the S. etruscus EVE, Ixodes scapularis EVE and South Bay virus nucleoprotein (Fig. 4b). [file 41564_2024_1825_MOESM7_ESM.zip › Source_Data_Figure_4/Ixodes_scapularis547_24recycles_amber_9dc3a/Ixodes_scapularis547_24recycles_amber_9dc3a_pae.png]

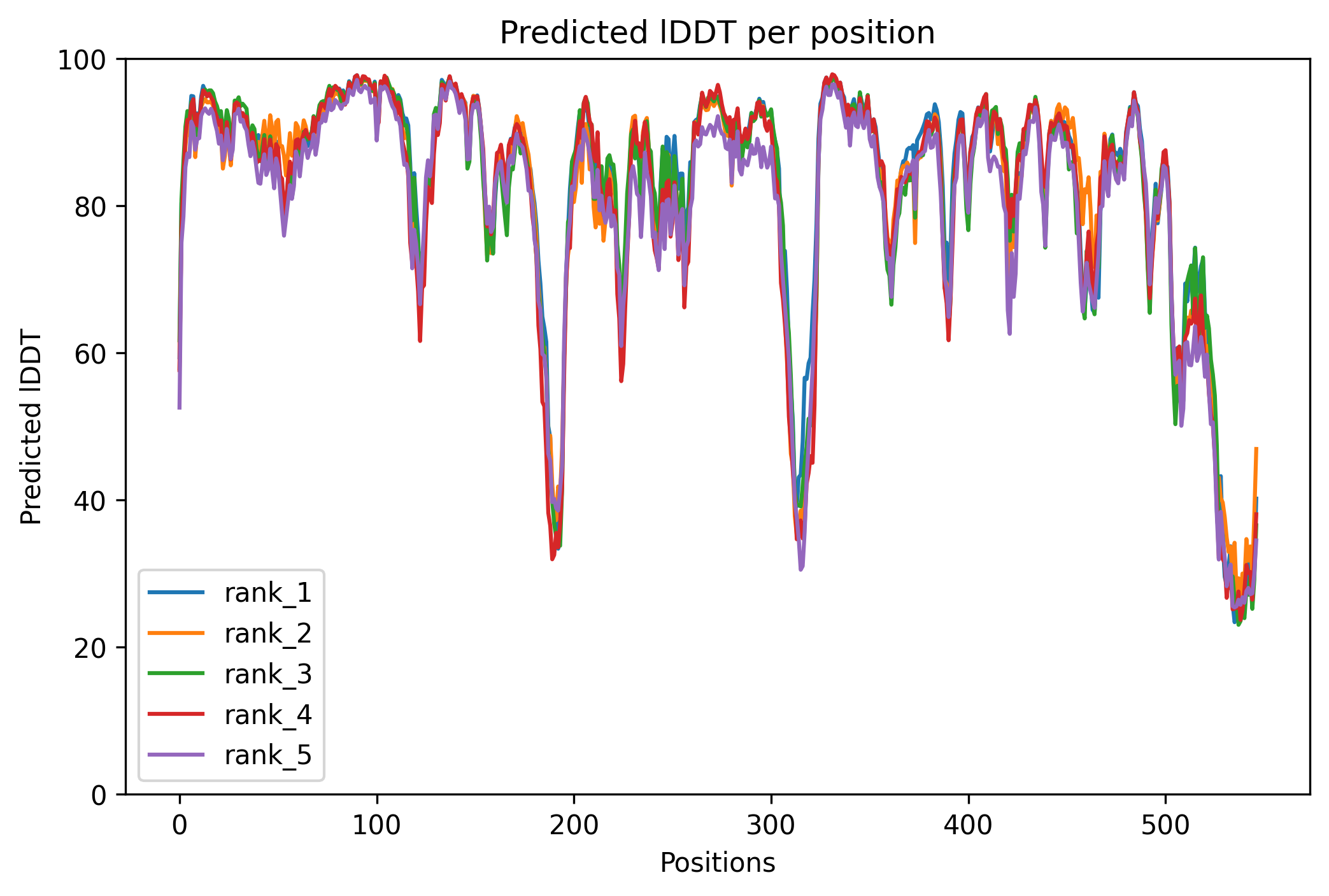

Supplement: Supplementary file 7 — NP tree files (Fig. 4a), and structural modelling files for the S. etruscus EVE, Ixodes scapularis EVE and South Bay virus nucleoprotein (Fig. 4b). [file 41564_2024_1825_MOESM7_ESM.zip › Source_Data_Figure_4/South_Bay_virus_48recycles_amber_b1f4e/South_Bay_virus_48recycles_amber_b1f4e_plddt.png]

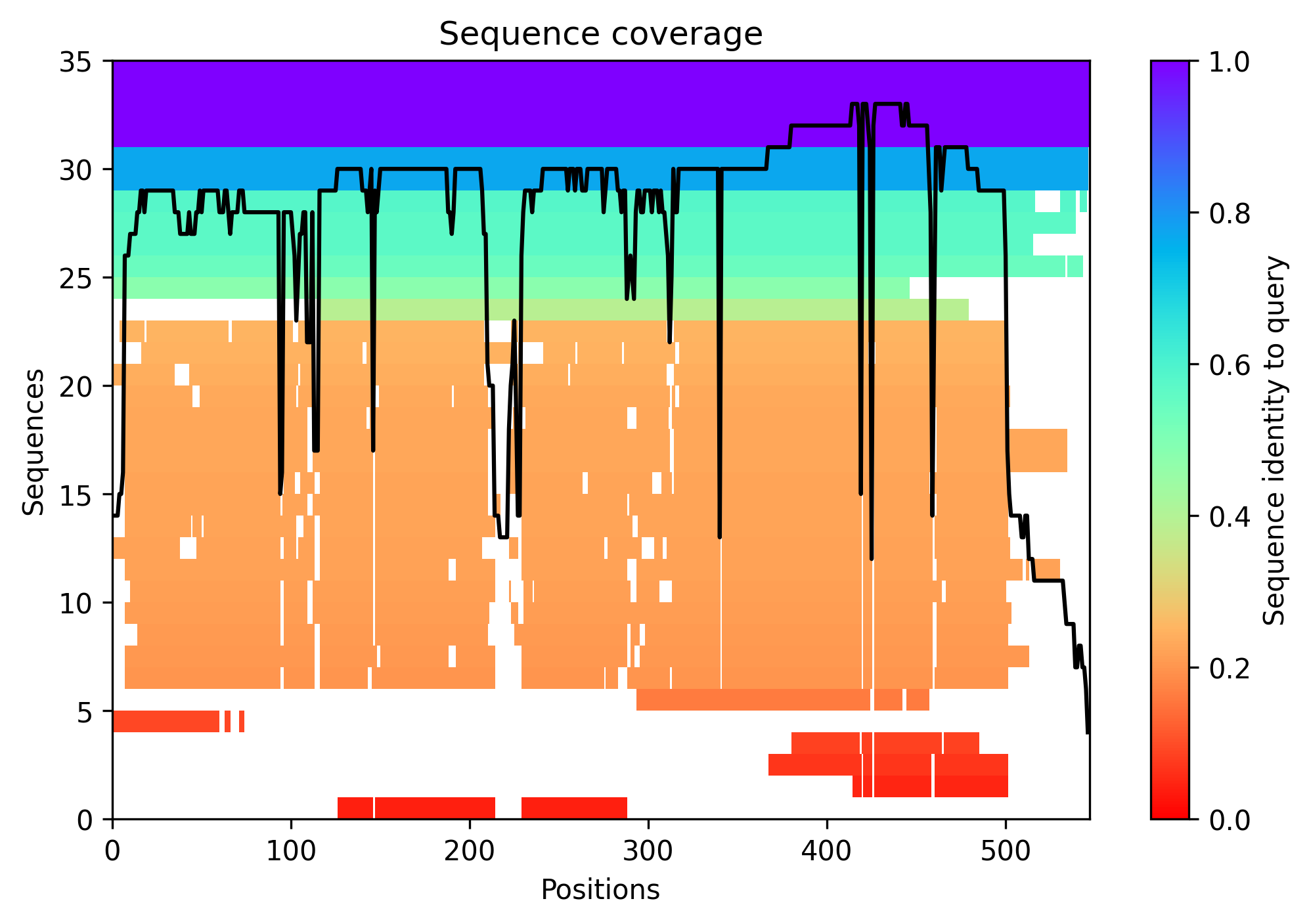

Supplement: Supplementary file 7 — NP tree files (Fig. 4a), and structural modelling files for the S. etruscus EVE, Ixodes scapularis EVE and South Bay virus nucleoprotein (Fig. 4b). [file 41564_2024_1825_MOESM7_ESM.zip › Source_Data_Figure_4/South_Bay_virus_48recycles_amber_b1f4e/South_Bay_virus_48recycles_amber_b1f4e_coverage.png]

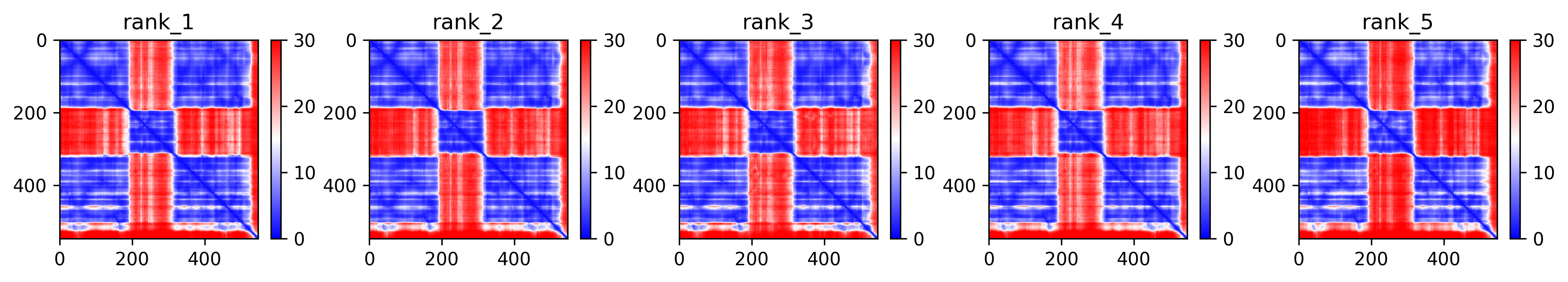

Supplement: Supplementary file 7 — NP tree files (Fig. 4a), and structural modelling files for the S. etruscus EVE, Ixodes scapularis EVE and South Bay virus nucleoprotein (Fig. 4b). [file 41564_2024_1825_MOESM7_ESM.zip › Source_Data_Figure_4/South_Bay_virus_48recycles_amber_b1f4e/South_Bay_virus_48recycles_amber_b1f4e_pae.png]

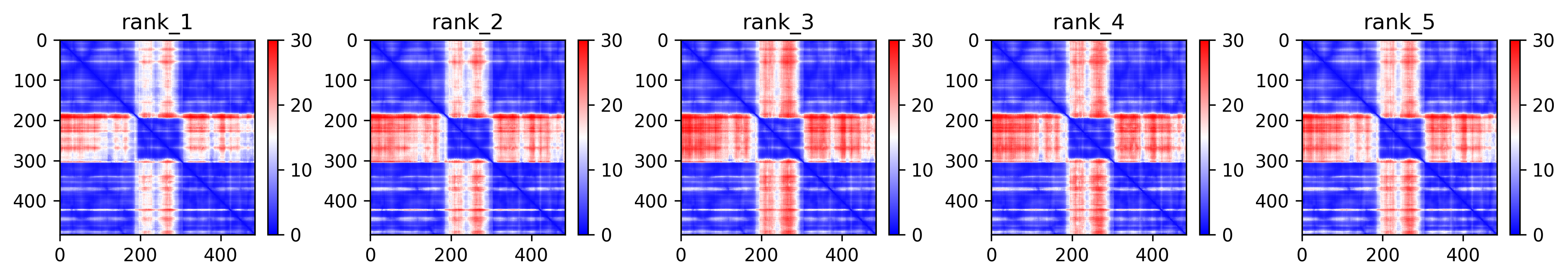

Supplement: Supplementary file 7 — NP tree files (Fig. 4a), and structural modelling files for the S. etruscus EVE, Ixodes scapularis EVE and South Bay virus nucleoprotein (Fig. 4b). [file 41564_2024_1825_MOESM7_ESM.zip › Source_Data_Figure_4/Suncus_etruscus_colabfold_24recycles_amber_8234a/Suncus_etruscus_colabfold_24recycles_amber_8234a_pae.png]

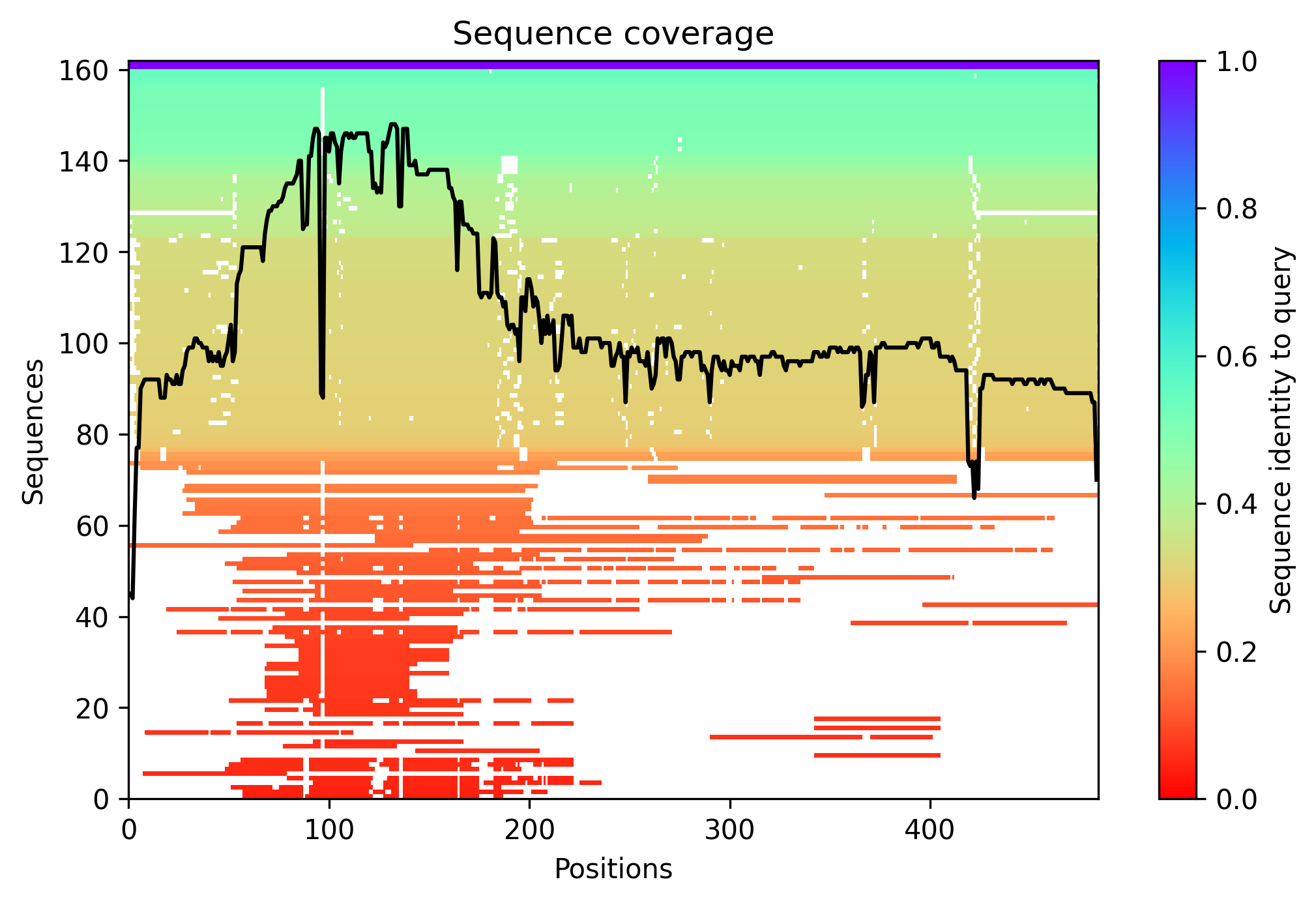

Supplement: Supplementary file 7 — NP tree files (Fig. 4a), and structural modelling files for the S. etruscus EVE, Ixodes scapularis EVE and South Bay virus nucleoprotein (Fig. 4b). [file 41564_2024_1825_MOESM7_ESM.zip › Source_Data_Figure_4/Suncus_etruscus_colabfold_24recycles_amber_8234a/Suncus_etruscus_colabfold_24recycles_amber_8234a_coverage.png]

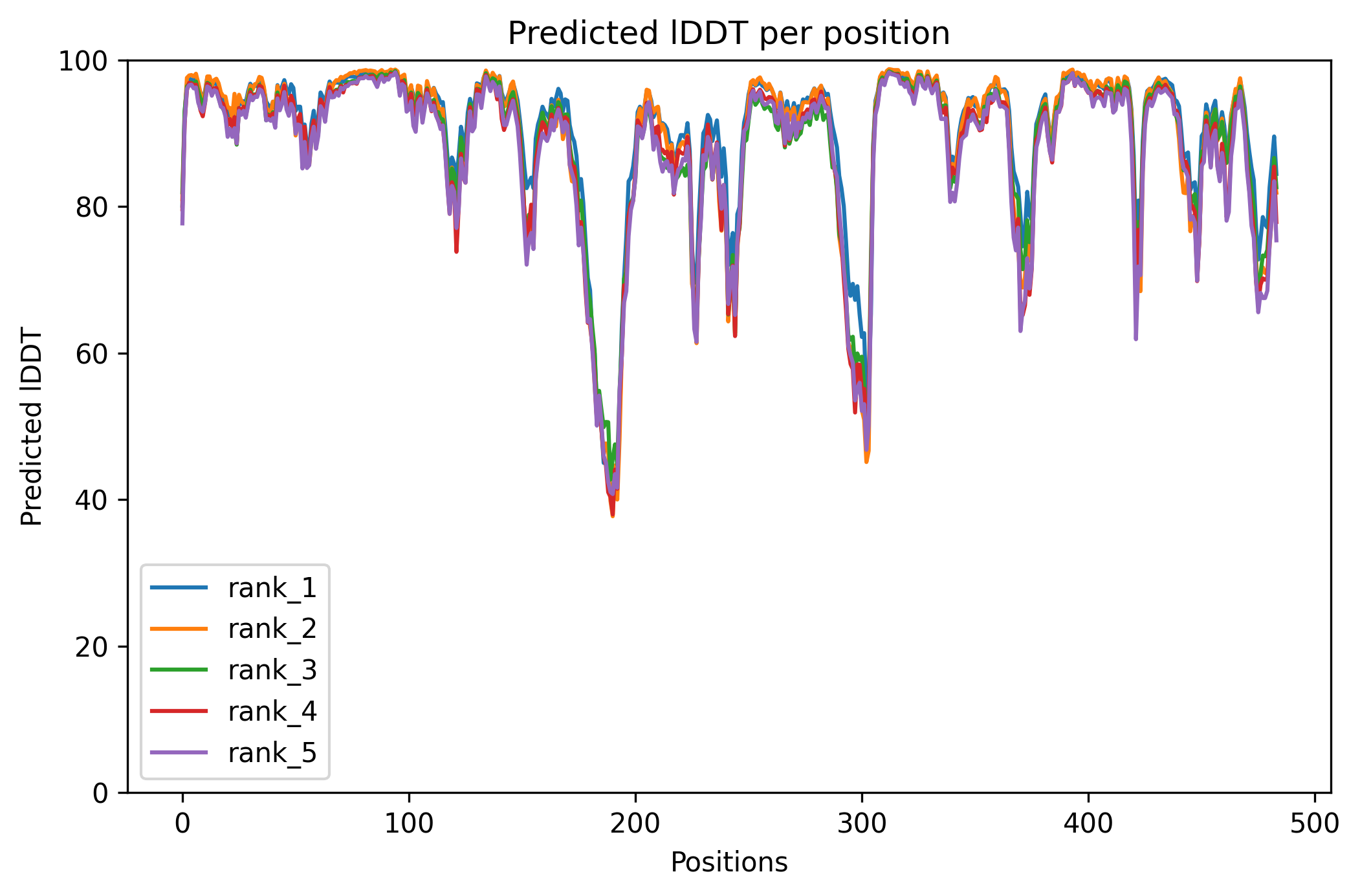

Supplement: Supplementary file 7 — NP tree files (Fig. 4a), and structural modelling files for the S. etruscus EVE, Ixodes scapularis EVE and South Bay virus nucleoprotein (Fig. 4b). [file 41564_2024_1825_MOESM7_ESM.zip › Source_Data_Figure_4/Suncus_etruscus_colabfold_24recycles_amber_8234a/Suncus_etruscus_colabfold_24recycles_amber_8234a_plddt.png]
